# Performance and Cost-Effectiveness of Computed Tomography Lung Cancer Screening Scenarios in a Population-Based Setting: A Microsimulation Modeling Analysis in Ontario, Canada

**DOI:** 10.1371/journal.pmed.1002225

**Published:** 2017-02-07

**Authors:** Kevin ten Haaf, Martin C. Tammemägi, Susan J. Bondy, Carlijn M. van der Aalst, Sumei Gu, S. Elizabeth McGregor, Garth Nicholas, Harry J. de Koning, Lawrence F. Paszat

**Affiliations:** 1 Department of Public Health, Erasmus MC—University Medical Center Rotterdam, Rotterdam, the Netherlands; 2 Department of Health Sciences, Brock University, St. Catharines, Ontario, Canada; 3 University of Toronto Dalla Lana School of Public Health, Ontario, Canada; 4 Institute for Clinical Evaluative Sciences, Ontario Tobacco Research Unit, Toronto, Ontario, Canada; 5 Institute for Clinical Evaluative Sciences, Toronto, Ontario, Canada; 6 Population, Public & Indigenous Health, Alberta Health Services, Calgary, Alberta, Canada; 7 The Ottawa Hospital Research Institute, Ottawa, Ontario, Canada; University of Pittsburgh, UNITED STATES

## Abstract

**Background:**

The National Lung Screening Trial (NLST) results indicate that computed tomography (CT) lung cancer screening for current and former smokers with three annual screens can be cost-effective in a trial setting. However, the cost-effectiveness in a population-based setting with >3 screening rounds is uncertain. Therefore, the objective of this study was to estimate the cost-effectiveness of lung cancer screening in a population-based setting in Ontario, Canada, and evaluate the effects of screening eligibility criteria.

**Methods and Findings:**

This study used microsimulation modeling informed by various data sources, including the Ontario Health Insurance Plan (OHIP), Ontario Cancer Registry, smoking behavior surveys, and the NLST. Persons, born between 1940 and 1969, were examined from a third-party health care payer perspective across a lifetime horizon. Starting in 2015, 576 CT screening scenarios were examined, varying by age to start and end screening, smoking eligibility criteria, and screening interval. Among the examined outcome measures were lung cancer deaths averted, life-years gained, percentage ever screened, costs (in 2015 Canadian dollars), and overdiagnosis. The results of the base-case analysis indicated that annual screening was more cost-effective than biennial screening. Scenarios with eligibility criteria that required as few as 20 pack-years were dominated by scenarios that required higher numbers of accumulated pack-years. In general, scenarios that applied stringent smoking eligibility criteria (i.e., requiring higher levels of accumulated smoking exposure) were more cost-effective than scenarios with less stringent smoking eligibility criteria, with modest differences in life-years gained. Annual screening between ages 55–75 for persons who smoked ≥40 pack-years and who currently smoke or quit ≤10 y ago yielded an incremental cost-effectiveness ratio of $41,136 Canadian dollars ($33,825 in May 1, 2015, United States dollars) per life-year gained (compared to annual screening between ages 60–75 for persons who smoked ≥40 pack-years and who currently smoke or quit ≤10 y ago), which was considered optimal at a cost-effectiveness threshold of $50,000 Canadian dollars ($41,114 May 1, 2015, US dollars). If 50% lower or higher attributable costs were assumed, the incremental cost-effectiveness ratio of this scenario was estimated to be $38,240 ($31,444 May 1, 2015, US dollars) or $48,525 ($39,901 May 1, 2015, US dollars), respectively. If 50% lower or higher costs for CT examinations were assumed, the incremental cost-effectiveness ratio of this scenario was estimated to be $28,630 ($23,542 May 1, 2015, US dollars) or $73,507 ($60,443 May 1, 2015, US dollars), respectively.

This scenario would screen 9.56% (499,261 individuals) of the total population (ever- and never-smokers) at least once, which would require 4,788,523 CT examinations, and reduce lung cancer mortality in the total population by 9.05% (preventing 13,108 lung cancer deaths), while 12.53% of screen-detected cancers would be overdiagnosed (4,282 overdiagnosed cases). Sensitivity analyses indicated that the overall results were most sensitive to variations in CT examination costs. Quality of life was not incorporated in the analyses, and assumptions for follow-up procedures were based on data from the NLST, which may not be generalizable to a population-based setting.

**Conclusions:**

Lung cancer screening with stringent smoking eligibility criteria can be cost-effective in a population-based setting.

## Abstract 2

In a microsimulation modelling analysis of a population in Ontario, Canada, Kevin ten Haaf and colleagues compare 576 different lung cancer screening policies with different screening frequencies and eligibility criteria.

## Author Summary

**Why Was This Study Done?:**

- In the US, lung cancer screening is recommended for current and former smokers who have quit within the past 15 y, aged 55 through 80 who smoked at least 30 pack-years; other countries are investigating the feasibility of implementing lung cancer screening policies.
- Despite lung cancer screening being recommended by a number of organizations, the cost-effectiveness of lung cancer screening is uncertain; concerns have been raised on the potential costs of implementing lung cancer screening.
- Past studies that evaluated the cost-effectiveness of lung cancer screening yielded inconclusive results. However, these studies considered limited numbers of screening policies, providing limited information on how different screening policy characteristics affect the cost-effectiveness of lung cancer screening.

**What Did the Researchers Do and Find?:**

- This study investigated how different screening policy characteristics, such as screening starting and stopping ages, screening interval, and different smoking history eligibility criteria, influence the performance and cost-effectiveness of lung cancer screening.
- A microsimulation model was used to analyze 576 different lung cancer screening policies for persons born between 1940 and 1969 in Ontario, Canada.
- The study found that requiring stringent smoking history eligibility criteria (i.e., requiring higher levels of accumulated smoking exposure) was more cost-effective than less stringent smoking history eligibility criteria.

**What Do These Findings Mean?:**

- Limiting screening to individuals with substantial (past) smoking histories may allow lung cancer screening to be implemented in a cost-effective manner.
- In contrast to initial expectations, annual screening is suggested to be more cost-effective than biennial screening.

## Introduction

The National Lung Screening Trial (NLST) showed that screening with low-dose computed tomography (CT) can reduce lung cancer mortality [1]. Although the sensitivity of CT screening in the NLST was reported to be over 90% across the three screening rounds, the reported specificity ranged from 73.4% in the first round to 83.9% in the third round [1]. Overall, 23.3% of the CT screens in the NLST were false positive, which often required additional follow-up CT examinations and, infrequently, invasive procedures (such as a biopsy, bronchoscopy, or thoracotomy) to determine the malignancy of one or more suspicious pulmonary nodules detected by CT screening [1]. Lung cancer screening with three annual screens, as performed in the NLST, was reported to be cost-effective by US standards, yielding estimated cost-effectiveness ratios of US$52,000 per life-year gained and US$81,000 per quality-adjusted life-year gained [2,3]. However, although the cost-effectiveness of lung cancer screening in a population-based setting has been examined previously, it has not been examined extensively [4–9].

To determine the cost-effectiveness of implementing cancer screening programs, microsimulation modeling is invaluable [10,11]. The United States Preventive Services Task Force (USPSTF) recommended lung cancer screening for current and former smokers who have quit within the past 15 y, aged 55 through 80 who smoked at least 30 pack-years [12]. This recommendation was in part based on a comparative modeling study using microsimulation models, as modeling allows one to extrapolate the results of randomized clinical trials and provide information on the long-term benefits and harms for screening programs with different designs and populations than those considered in clinical trials [13]. However, although the modeling study that informed the USPSTF provides an understanding of the trade-offs between the benefits and harms of different screening scenarios, it did not formally consider their cost-effectiveness [13].

In Ontario, Canada, lung cancer is responsible for the largest proportion of cancer deaths (49.9 per 100,000 individuals) in the population of 13.8 million individuals, despite falling smoking rates [15,28,55,56]. The implementation of a lung cancer screening program, in addition to continued efforts in primary prevention of smoking, could reduce lung cancer mortality. However, concerns have been raised about whether and how such a program can be implemented in a cost-effective manner [14,16]. Previous studies on the cost-effectiveness of population-based lung cancer screening have yielded inconclusive results, ranging from US$18,452–US$66,480 per life-year gained and US$27,756–US$243,077 per quality-adjusted life-year gained [4–9]. However, many of these studies reported the average cost-effectiveness ratios (ACER, the ratio of differences in costs to differences in health effects compared to no screening) of the investigated screening scenarios as the incremental cost-effectiveness ratios (ICER, the ratio of incremental costs to incremental health effects of a screening policy relative to its next best alternative), which can give misleading cost-effectiveness estimates [17].

Furthermore, these studies considered limited numbers of screening scenarios, providing little information on the effects of screening eligibility criteria, and may have had insufficient numbers of comparator scenarios to yield correct ICERs [18]. The aim of this study was to investigate the benefits (such as lung cancer mortality reduction and the number of life-years gained), harms (such as the number of false-positive results and occurrence of overdiagnosis), and cost-effectiveness of many different lung cancer screening scenarios for the population of Ontario, overcoming some of the limitations of previous studies.

## Materials and Methods

### Ethics Statement

This study was approved by the Research Ethics Board of Sunnybrook Health Sciences Centre on behalf of the Institute for Clinical Evaluative Sciences (ICES). Individual consent for access to de-identified data was not required.

### MISCAN-Lung

The MIcrosimulation SCreening ANalysis (MISCAN) Lung model was used for this analysis. Other versions of the MISCAN model have been used to investigate the cost-effectiveness of screening programs for breast, colorectal, cervical, and prostate cancers [19–22]. The MISCAN-Lung model used in these analyses was previously calibrated to individual-level data from the NLST and the Prostate, Lung, Colorectal, and Ovarian Cancer Screening Trial, from which information on the preclinical duration of lung cancer and the effectiveness of CT screening were derived [23,24]. MISCAN-Lung was one of the models used to inform the USPSTF on their recommendations for lung cancer screening [13]. The structure of the model and its underlying assumptions have been described previously and are detailed in S1 Text; the characteristics of the investigated population are described in the following section and in S2 and S3 Texts [23,24].

In brief, MISCAN-Lung simulates life histories for each individual in the considered population from birth until death in the presence and absence of screening. For each individual, a smoking history is generated based on data on the investigated population. A person’s smoking history influences the probability of developing preclinical lung cancer as well as the probability of dying from other causes. The model considers four histological types of lung cancer: adenocarcinoma/large cell carcinoma/bronchioloalveolar carcinoma, squamous cell carcinoma, other non-small cell carcinoma, and small cell carcinoma. Once preclinical lung cancer has developed, it is assumed to progress through stages IA to IV. During each stage, the cancer may be detected due to symptoms, after which the person is assumed to undergo treatment with associated treatment costs. Lung cancer survival after clinical diagnosis is dependent on the histology and stage of the cancer and the person’s gender. If the screening component is activated, preclinical lung cancers may be detected by screening (at the expense of screening-related costs), which may alter a person’s life history: detection by screening allows treatment at an earlier stage, which may cure the individual, allowing him or her to resume a normal (lung cancer-free) life history. The probability that an individual is cured due to early detection differs by the stage at detection. Screening may also result in serious harms, such as overdiagnosis (the detection of a disease that would never have been detected if screening had not occurred), which may lead to unnecessary (invasive) follow-up procedures, treatments, and anxiety. The effects of screening are derived through utilizing information on the preclinical duration of lung cancer, the screen-detectability of lung cancer, and relevant information on the examined population (such as smoking behavior and other-cause mortality corrected for smoking history) to model the life histories of individuals in the presence and absence of screening [25]. Through comparing the life histories in the presence of screening with the corresponding life histories in the absence of screening, MISCAN-Lung can estimate the effectiveness and costs of screening scenarios.

### Simulated Population

Three different birth cohorts were investigated: 1940–1949 (ages 66–75 in 2015), 1950–1959 (ages 56–65 in 2015), and 1960–1969 (ages 46–55 in 2015). These cohorts represent approximately 5.2 million individuals in 2016 in the age range for which the USPSTF currently recommends lung cancer screening [12,26]. Birth tables for each cohort were derived from information from Statistics Canada and the Ontario Ministry of Finance [26,27]. Ontario-specific data on smoking behavior were used to model smoking initiation and cessation probabilities and the average number of cigarettes smoked per day (divided into five categories) by age and gender for each cohort [28–32]. Life tables by birth year and gender were extracted from the Canadian Human Mortality Database and adjusted for smoking behavior and lung cancer mortality, as shown graphically for persons born in 1955 in S2 Text Figs C–F [32]. Further information on the methods used to model smoking behavior and adjustment of the life tables for smoking behavior is provided in S2 Text. The age- and gender-specific lung cancer incidence, histology proportions, and stage proportions estimated by MISCAN-Lung were compared to observed data from the Ontario Cancer Registry from 2007–2009, in which screening did not occur, and are detailed in S3 Text [33].

### Screening Scenarios

In total, 576 potential screening scenarios were evaluated. The evaluated scenarios considered different combinations of the following characteristics: age to start screening; age to stop screening; screening interval; and screening eligibility regarding cumulative smoking exposure, years since smoking cessation (for former smokers, defined as individuals who have quit smoking permanently), and whether or not former smokers were excluded from further screening after they reach a maximum number of years since cessation (Table 1). Two types of cumulative smoking criteria, of which one is used at a time in an evaluated scenario, were distinguished: the first type was based on the cumulative number of pack-years, used in the NLST (“NLST-like”) [1]; the second type of cumulative smoking criteria was based on the criteria used in the Dutch-Belgian lung cancer screening trial (NELSON), which evaluated a person’s smoking duration and average number of cigarettes per day separately (“NELSON-like”) [34]. Of the 576 screening scenarios, 216 screening scenarios considered “NLST-like” criteria (including the criteria currently recommended by the USPSTF), whereas 360 screening scenarios considered “NELSON-like” criteria. Perfect attendance to screening was assumed for the base-case investigation. Estimations regarding screen-related procedures such as false-positive results (defined as receiving a positive screening test result when lung cancer is not found after diagnostic work-up), follow-up CT examinations, screen-related biopsies/bronchoscopies, and non-lung cancer-related surgeries were derived from individual-level data from the CT-arm of the NLST and are described in S4 Text. Limited information on morbidity and mortality has been reported from randomized controlled trials. In a subgroup analysis of NELSON participants, 1% of participants were found to have incidental findings that required additional work-up procedures or treatment [35]. However, no information on morbidity or mortality was reported. In the NLST, 0.06% of the positive screening tests in the low-dose CT group that did not result in a diagnosis of lung cancer were associated with a major complication after an invasive procedure [1]. Overall, six individuals with a positive screening test in the low-dose CT group that did not result in a diagnosis of lung cancer died within 60 d after an invasive diagnostic procedure (0.04%), but it was unknown whether these deaths were caused by complications of the diagnostic procedures [1]. Thus, given the low occurrence of invasive procedures along with a low frequency of major complications, the occurrence of morbidity or death related to screen-related follow-up procedures is expected to be minor.

**Table 1:** Characteristics of the lung cancer screening scenarios evaluated by the MISCAN-Lung model.

Scenario characteristic	Considered values
Age to start screening	50, 55, 60
Age to stop screening	75, 80
Screening interval	Annual, Biennial
Minimum cumulative smoking criteria*	
Pack-years (NLST-like scenarios)	20 pack-years, 30 pack-years, 40 pack-years
Minimum number of years smoked and minimum number of cigarettes per day during years smoked (NELSON-like scenarios)	25 y of smoking at least 10 cigarettes per day **or** 30 y of smoking at least 5 cigarettes per day
	20 y of smoking at least 15 cigarettes per day **or** 25 y of smoking at least 10 cigarettes per day
	25 y of smoking at least 15 cigarettes per day **or** 30 y of smoking at least 10 cigarettes per day
	30 y of smoking at least 15 cigarettes per day **or** 35 y of smoking at least 10 cigarettes per day
**Additional smoking-related criteria**	
Maximum number of years since smoking cessation to be eligible for first screening invitation	10, 15, 20
Exclusion from further screening after reaching the maximum number of years since smoking cessation	No, Yes

*Either pack-years or minimum number of years smoked and minimum number of cigarettes per day during years smoked are used in an evaluated screening scenario.

### Costs

The analyses were conducted from a third-party health care payer perspective. Fully allocated costs for lung cancer treatment were estimated by stage, age, and gender from the date of diagnosis until the date of death or last known date of follow-up (by person-month) through data from the Ontario Health Insurance Plan (OHIP), the Canadian Institute for Health Information (CIHI), the Ontario Drug Benefit Plan database, the Ontario Chronic Care database, the Ontario Home Care database, and the Ontario New Drug Funding Program. These datasets were linked using unique encoded identifiers and were analyzed at the ICES. Controls without a lung cancer diagnosis from the Registered Persons Database (a roster of all OHIP beneficiaries) were matched to 12,713 staged cases of lung cancer from the Ontario Cancer Registry (10 controls matched per case), based on age, sex, median household income, and census tract on the date of diagnosis of the case. Fully allocated costs were estimated similarly for controls. The fully allocated costs of controls were subtracted from the fully allocated costs for cases in order to obtain the attributable costs of lung cancer care by phase of care (initial, continuing, and terminal care) [36]. By incorporating the fully allocated costs of lung cancer care by phase of care, it is taken into account that individuals whose lung cancer death is averted will on average incur higher costs over their remaining lifetime. In MISCAN-Lung, the attributable costs for stage I were assumed for the modeled stages IA and IB, whereas the attributable costs of stage III were assumed for the modeled stages IIIA and IIIB.

Each person’s eligibility for lung cancer screening was assumed to be free of misclassification error. Therefore, upon entering the eligible age range for the considered screening scenarios, each ever-smoking individual was assumed to receive an invitation for a lung cancer risk assessment. It was assumed that half of all ever-smokers would accept this invitation; half of the individuals who participated in the risk assessment were assumed to request a consultation with a primary care physician about their risk. Costs for screening-related events were determined using 2013 data from OHIP and CIHI. The costs for screening invitations and fixed costs related to the screening program, such as costs for the screening registry, program infrastructure, communications, and advertising, were derived from those incurred in the recent establishment of the colorectal screening program administered by Cancer Care Ontario. The costs for risk assessments were estimated assuming that screening program staff trained in health communication would administer the assessments. Fixed costs were counted up to the year in which the last individuals in the cohorts are eligible for screening (2045 for screening scenarios that end at age 75, 2050 for screening scenarios that end at age 80).

All costs were expressed in Canadian dollars (using May 2013 levels as a base) and were adjusted to reflect the May 2015 prices for health care services using the Ontario Consumer Price Index derived from Statistics Canada [37]. A lifetime time horizon for the costs and effects of screening was applied to each simulated person. Annual discount rates of 3% were applied to both costs and effects, using 2015 as the reference year [38]. The estimated attributable costs of lung cancer care by phase of care and the estimated costs related to the screening program are presented in Tables 2 and 3, respectively. To reflect the uncertainty in these cost estimates, sensitivity analyses were performed, which varied the costs by 50%, as described in a later section of this manuscript. Although cost-effectiveness thresholds have been proposed in the past, there is no official cost-effectiveness threshold employed in the Canadian health care system [37]. Therefore, a cost-effectiveness threshold of $50,000 Canadian dollars ($41,114 in May 1, 2015, US dollars) per life-year gained was chosen, similar to previous Canadian cost-effectiveness studies [39,40].

**Table 2:** Attributable costs (in Canadian dollars) estimates used in the MISCAN-Lung model. Costs were estimated per phase of lung cancer care per person-year of treatment by gender, age, and stage of disease for Ontario, Canada.

**Initial care phase**	**Men**					**Women**			
**Age group/Lung cancer stage**	**Stage I**	**Stage II**		**Stage III**	**Stage IV**	**Stage I**	**Stage II**	**Stage III**	**Stage IV**
**Younger than 60**	$30,272	$41,283		$49,676	$45,374	$27,172	$42,441	$44,576	$43,170
**60–69**	$29,440	$37,959		$45,788	$38,073	$27,454	$37,773	$47,589	$39,411
**70–79**	$31,603	$39,444		$41,832	$32,659	$27,178	$43,439	$42,779	$32,493
**Older than 80**	$27,391	$24,669		$32,569	$29,886	$27,284	$32,394	$29,414	$29,035
**Continuing care phase**	**Men**					**Women**			
**Age group/Lung cancer stage**	**Stage I**	**Stage II**		**Stage III**	**Stage IV**	**Stage I**	**Stage II**	**Stage III**	**Stage IV**
**Younger than 60**	$5,617	$8,451		$10,458	$18,683	$4,859	$6,777	$8,626	$13,716
**60–69**	$3,371	$4,276		$7,270	$5,004	$5,559	$7,424	$9,214	$12,062
**70–79**	$3,019	$1,938		$5,011	$5,616	$2,124	$6,447	$6,999	$5,656
**Older than 80**	$4,785	$1,938		$1,689	$5,616	$2,022	$6,447	$7,475	$10,882
**Terminal care phase (death due to causes other than lung cancer)**	**Men**					**Women**			
**Age group/Lung cancer stage**	**Stage I**	**Stage II**		**Stage III**	**Stage IV**	**Stage I**	**Stage II**	**Stage III**	**Stage IV**
**Younger than 60**	$17,174	$21,061		$17,775	$23,884	$13,741	$2,332	$4,689	$14,284
**60–69**	$13,596	$10,586		$13,690	$17,548	$13,741	$20,660	$5,591	$6,869
**70–79**	$15,887	$15,887		$9,858	$9,032	$15,875	$10,999	$12,917	$4,300
**Older than 80**	$15,887	$15,887		$9,368	$8,243	$21,554	$28,188	$2,217	$6,030
**Terminal care phase of lung cancer (lung cancer death)**	**Men**					**Women**			
**Age group/Lung cancer stage**	**Stage I**	**Stage II**	**Stage III**		**Stage IV**	**Stage I**	**Stage II**	**Stage III**	**Stage IV**
**Younger than 60**	$72,167	$70,323	$84,041		$98,611	$51,164	$71,024	$89,322	$94,906
**60–69**	$73,085	$82,296	$84,828		$95,000	$51,164	$81,256	$79,563	$87,113
**70–79**	$68,187	$91,114	$77,067		$97,320	$68,844	$73,176	$77,424	$89,056
**Older than 80**	$55,413	$141,182	$69,807		$78,002	$68,844	$59,590	$83,353	$73,453

Costs were estimated from a third-party health care payer perspective by matching lung cancer patients in the Ontario Cancer Registry to persons registered in the OHIP, free of lung cancer, by age, sex, median household income, and census tract on the date of diagnosis of the case. Additionally, data from the CIHI, the Ontario Drug Benefit Plan database, the Ontario Chronic Care database, the Ontario Home Care database, and the Ontario New Drug Funding Program were used.

**Table 3:** Costs (in Canadian dollars) of screening-related events and fixed program costs used in the MISCAN-Lung model.

**Cost estimates related to screening invitations**	
**Description**	**Unit costs**
Invitation to assess lung cancer risk	$5
Lung cancer risk assessment	$32
Visit with primary care physician with regards to lung cancer risk assessment	$67
Initial and repeat screening invitations	$3
**Cost estimates for screening- and follow-up–related procedures**	
**Description**	**Unit costs**
Screening CT examination	$430
Follow-up CT examination	$430
Visit with primary care physician with regards to the results of a follow-up chest CT	$41
Percutaneous cytologic analysis/bronchoscopy/biopsy	$1,355
Non-lung cancer surgery for potentially benign disease	$11,844
Fixed program cost estimates per year (per 100,000 individuals alive in 2015)*	
**Description**	**Yearly costs**
First year	$823,321
Second year up to the year in which the last individuals in the cohorts are eligible for screening (2045 for screening scenarios that end at age 75, 2050 for screening scenarios that end at age 80)	$411,660

Costs for screening-related events were estimated using 2013 data from the OHIP and the CIHI. The costs for program invitations and fixed costs related to the screening program, such as costs for the screening registry, program infrastructure, communications, and advertising, were derived from those incurred in the recent establishment of ColonCancerCheck, the colorectal screening program administered by Cancer Care Ontario. The costs for lung cancer risk assessments were estimated assuming that screening program staff trained in health communication would administer the assessments.

*The fixed costs per 100,000 individuals alive in 2015 consist of one-time, first year only startup costs for Information Technology infrastructure ($411,661 Canadian dollars), annual maintenance costs for Information Technology infrastructure ($61,749 Canadian dollars), annual costs for maintaining main screening centers ($144,081 Canadian dollars), annual costs for communications and advertising ($102,915 Canadian dollars), and annual costs for provincial program management and evaluation ($102,915 Canadian dollars).

### Benefits, Harms, and Cost-Effectiveness of Screening Scenarios

For each screening scenario, the number of lung cancer deaths prevented, life-years gained, proportion of individuals ever screened, number of CT examinations, screen-related biopsies/bronchoscopies, false-positive screens, non-lung cancer-related surgeries, overdiagnoses, and costs were compared with a situation in which screening does not occur, from 2015 onward. Screening scenarios that were more costly and less effective (i.e., fewer life-years gained) than other scenarios were ruled out as non-efficient by simple dominance. Scenarios that were more costly and less effective than a combination of other scenarios were also ruled out as non-efficient by extended dominance. The remaining screening scenarios constitute the frontier of efficient screening scenarios, i.e., the efficient frontier. For each efficient screening scenario, the ICER was determined, calculated as the incremental net costs per incremental life-year gained compared to the previous efficient screening scenario.

### Sensitivity Analyses

A number of sensitivity analyses were performed to investigate which groups of cost estimates and attendance assumptions have the greatest influence on the cost-effectiveness estimates by varying the costs for CT examinations by 50% compared with the base-case analyses, varying the attributable costs of lung cancer care by phase of care by 50% compared with the base-case analyses, and imperfect attendance rates for screening: low attendance (33% overall compliance rate), average attendance (55% overall compliance rate), and high attendance (64% overall compliance rate). Sensitivity analyses were performed for all 576 scenarios to investigate the effects of variations in assumptions on the composition of the efficient frontier.

## Results

### Screening Scenarios on the Efficient Frontier

**Fig 1:**
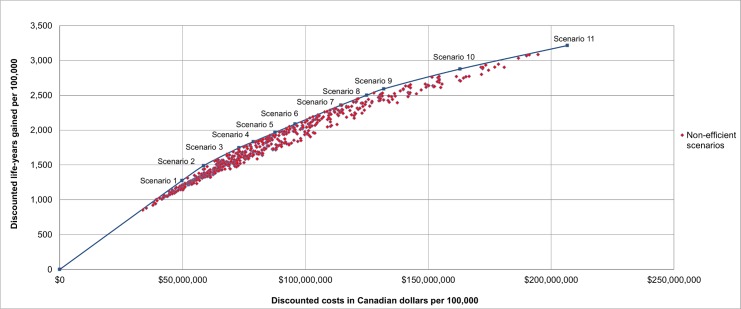
The cost-effectiveness of all 576 investigated lung cancer screening scenarios in the base-case analysis. Results are presented per 100,000 individuals alive in 2015 and are discounted by 3% annually. Scenarios on the efficient frontier are described in Table 4.

**Fig 2:**
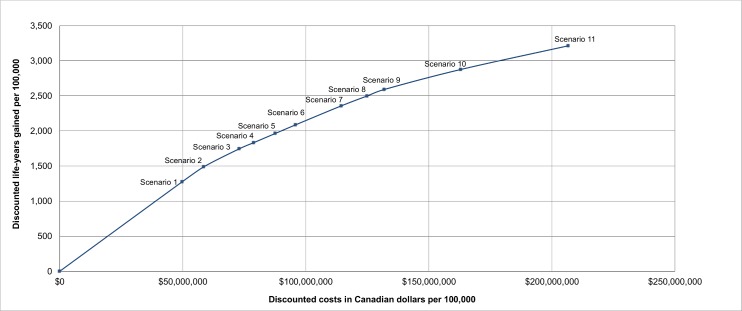
The incremental cost-effectiveness of the lung cancer screening scenarios on the efficient frontier. Results are presented per 100,000 individuals alive in 2015 and are discounted by 3% annually. Scenarios on the efficient frontier are described in Table 4.

The net discounted costs and life-years gained (from 2015 onwards) for each scenario were used to determine the screening scenarios on the efficient frontier, i.e., the scenarios that provide the highest number of life-years gained for their costs, in the base-case analysis, as shown in Figs 1 and 2. The scenarios that are on the efficient frontier are described in Table 4 and shown in Fig 2. A complete overview of the net discounted costs and life-years gained of all investigated screening scenarios is presented in S1 Appendix Figs A–H. All outcomes were reported per 100,000 individuals alive in 2015.

**Table 4:** Cost-effectiveness estimates for lung cancer screening scenarios on the efficient frontier.

Scenario #	Starting age of screening	Stopping age of screening	Screening interval	Maximum number of years since cessation	Cumulative smoking criteria	Exclusion from further screening invitations after reaching the maximum number of years since cessation	Discounted costs compared to no screening (in Canadian dollars) per 100,000	Discounted life-years gained per 100,000	Costs (in Canadian dollars) per life-year gained (discounted)/ACER compared to no screening	ICER compared to the previous efficient scenario
#1	60	75	Annual	10	40 pack-years (NLST-like)	Yes	$49,768,886	1,276	$39,006	-
#2	55	75	Annual	10	40 pack-years (NLST-like)	Yes	$58,549,938	1,489	$39,311	$41,136
#3	55	75	Annual	10	30 pack-years (NLST-like)	Yes	$72,978,421	1,746	$41,801	$56,262
#4	55	80	Annual	10	30 pack-years (NLST-like)	Yes	$78,858,485	1,834	$43,001	$66,802
#5	55	75	Annual	15	30 pack-years (NLST-like)	Yes	$87,658,495	1,965	$44,600	$66,885
#6	55	80	Annual	15	30 pack-years (NLST-like)	Yes	$95,859,980	2,088	$45,916	$67,065
#7	55	80	Annual	20	30 y of smoking at least 15 cigarettes per day **or** 35 y of smoking at least 10 cigarettes per day (NELSON-like)	Yes	$114,462,449	2,359	$48,530	$68,675
#8	55	80	Annual	20	30 y of smoking at least 15 cigarettes per day **or** 35 y of smoking at least 10 cigarettes per day (NELSON-like)	No	$124,978,314	2,500	$49,998	$74,557
#9	50	80	Annual	20	30 y of smoking at least 15 cigarettes per day **or** 35 y of smoking at least 10 cigarettes per day (NELSON-like)	No	$131,929,978	2,592	$50,901	$75,370
#10	50	80	Annual	20	25 y of smoking at least 15 cigarettes per day **or** 30 y of smoking at least 10 cigarettes per day (NELSON-like)	No	$162,994,771	2,877	$56,661	$109,083
#11	50	80	Annual	20	25 y of smoking at least 10 cigarettes per day **or** 30 y of smoking at least 5 cigarettes per day (NELSON-like)	No	$206,703,139	3,214	$64,304	$129,394

Results are per 100,000 individuals alive at the start of 2015. An annual discount rate of 3% annually was applied to costs and life-years gained.

All scenarios on the efficient frontier consist of annual screening (Table 4), while biennial screening is dominated. Assuming a cost-effectiveness threshold of $50,000 Canadian dollars ($41,114 May 1, 2015, US dollars) per life-year gained as acceptable for the Canadian health care system, Scenario #2 was considered optimal: current and former smokers (who quit ≤ 10 y ago) who smoked ≥40 pack-years would be screened annually between ages 55–75, yielding an ICER of $41,136 Canadian dollars ($33,825 May 1, 2015, US dollars) per life-year gained. If 50% lower or higher attributable costs were assumed, the ICER of this scenario was estimated to be $38,240 ($31,444 May 1, 2015, US dollars) or $48,525 ($39,901 May 1, 2015, US dollars), respectively. If 50% lower or higher costs for CT examinations were assumed, the ICER of this scenario was estimated to be $28,630 ($23,542 May 1, 2015, US dollars) or $73,507 ($60,443 May 1, 2015, US dollars), respectively.

In addition, the benefits and harms of all scenarios on the efficient frontier were examined (Table 5). Scenario #2 would reduce lung cancer mortality in the overall population (which includes non-eligible individuals) by 9.05%, preventing 251 lung cancer deaths and gaining 2,531 life-years (undiscounted) over the lifetime of the program (i.e., on average, 10.08 life-years would be gained for each lung cancer death prevented). However, in Scenario #2, 9.56% of the overall population would receive at least one screen, requiring 91,692 CT screens and follow-up examinations. This scenario would lead to 14,729 false-positive screens and 163 surgeries for potentially benign disease (in persons in whom lung cancer is not detected) and 350 biopsies/bronchoscopies (in persons in whom lung cancer is not detected). Ultimately, 12.53% of all screen-detected cancers would be overdiagnosed, leading to 82 overdiagnosed cases. Based on the estimated number of individuals in the examined cohorts in 2016, Scenario #2 is estimated to screen 499,261 individuals at least once, require 4,788,523 CT examinations, and prevent 13,108 lung cancer deaths, while 4,282 cases of lung cancer would be overdiagnosed [26,27]. The average annual non-discounted costs compared to no screening would be approximately $1,400,000 Canadian dollars ($1,151,178 May 1, 2015, US dollars) over the considered time period; however, the annual costs are higher in the first years compared to later years, due to diminishing numbers of individuals meeting the eligibility criteria. For example, the average non-discounted costs compared to no screening are approximately $5,000,000 Canadian dollars ($4,111,350 May 1, 2015, US dollars) for 2015–2020 compared with approximately $1,600,000 Canadian dollars ($1,315,632 May 1, 2015, US dollars) in 2030–2035.

**Table 5:** Overview of selected benefits and harms (per 100,000 individuals alive at the start of 2015) of the screening scenarios on the efficient frontier (effect estimates are not discounted).

Scenario #*	Percentage of the total population ever screened	CT screens and follow-up examinations (per 100,000)	Lung cancer mortality reduction in the total population (%)	Lung cancer deaths prevented^†^ (per 100,000)	Life-years gained (per 100,000)	Average number of life-years gained per lung cancer death averted	Percentage of screen-detected cancers that are overdiagnosed	Number of overdiagnosed lung cancers (per 100,000)^‡^	False-positive screens (per 100,000)	Number of non-lung cancer surgeries due to screening^§^ (per 100,000)	Biopsies due to screening^§^ (per 100,000)
#1	8.74%	73,248	8.24%	229	2,170	9.48	13.06%	80	11,937	132	283
#2	9.56%	91,692	9.05%	251	2,531	10.08	12.53%	82	14,729	163	350
#3	13.03%	125,320	10.50%	292	2,993	10.25	12.31%	93	20,145	223	479
#4	13.04%	135,410	11.71%	325	3,159	9.72	14.43%	127	21,575	239	514
#5	15.41%	161,159	11.93%	331	3,388	10.24	12.34%	106	25,698	285	612
#6	15.42%	177,014	13.58%	377	3,624	9.61	14.68%	150	27,947	311	667
#7	16.06%	225,062	15.71%	436	4,129	9.47	14.89%	176	34,933	390	838
#8	16.06%	255,207	17.32%	481	4,422	9.19	15.48%	203	39,228	438	943
#9	16.19%	270,354	17.59%	489	4,577	9.36	15.36%	204	41,414	463	997
#10	19.99%	355,448	19.51%	542	5,142	9.49	15.15%	221	54,259	607	1,308
#11	26.13%	473,383	21.92%	609	5,774	9.48	15.14%	248	72,221	809	1,742

* Scenario details are provided in Table 4.

**†** Number of lung cancer deaths per 100,000 without screening: 2,777.

**‡** Number of lung cancer cases per 100,000 without screening: 3,522.

**§** For persons in whom lung cancer was not detected by screening.

### Effects of Screening Scenario Characteristics on Cost-Effectiveness

Scenarios with older starting ages have lower costs compared with scenarios that start at younger ages but also yield a smaller number of life-years gained (S1 Appendix Fig A). Raising the age to stop screening from 75 to 80 increases the costs and the number of life-years gained, but differences are modest (S1 Appendix Fig B). A comparison of scenarios by smoking eligibility criteria indicates that there is little difference between using NLST-like or NELSON-like smoking eligibility criteria (S1 Appendix Fig C).

S1 Appendix Figs D–G demonstrate the importance of cumulative smoking criteria on cost-effectiveness. Each increase in the cumulative smoking requirement for enrollment substantially decreases the costs while modestly decreasing the number of life-years gained in both NLST-like (S1 Appendix Fig D) and NELSON-like (S1 Appendix Fig E) screening scenarios. In other words, scenarios that apply stringent cumulative smoking eligibility criteria are closer to the efficient frontier than those that apply less restrictive cumulative smoking eligibility criteria. In general, scenarios that require only 20 pack-years are dominated by scenarios that apply more stringent (higher) pack-year criteria.

Increasing the maximum number of years since smoking cessation (S1 Appendix Fig F) and not excluding individuals from further screening once they reach the maximum number of years since cessation (S1 Appendix Fig G) both increase the costs and the numbers of life-years gained. However, the effects of these criteria are less pronounced than those related to cumulative smoking requirements.

S1 Appendix Fig H shows the effects of annual screening compared with biennial screening. Although biennial screening scenarios have substantially lower costs compared with annual screening scenarios, the life-years gained are also substantially lower. S1 Appendix Fig H demonstrates that annual screening scenarios dominate biennial screening scenarios.

### Sensitivity Analyses

**Fig 3:**
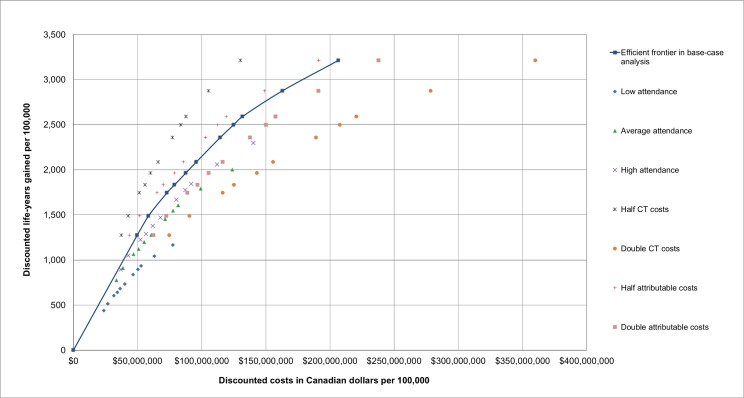
The incremental cost-effectiveness of the lung cancer screening scenarios on the efficient frontier and their corresponding cost-effectiveness throughout different sensitivity analyses. Results are presented per 100,000 individuals alive in 2015 and are discounted by 3% annually. The relative ranking of the scenarios is consistent across sensitivity analyses (i.e., if a scenario is more costly and gains more life-years than another scenario in the base-case analysis, this is also the case in all sensitivity analyses).

Altering assumptions about attendance rates, CT examination costs, and attributable costs impacted the scenarios on the efficient frontier in the base-case analysis to varying degrees. Fig 3 provides an overview of the scenarios on the efficient frontier in the base-case analysis along with the discounted life-years gained and costs for these scenarios in the sensitivity analyses.

Altering assumptions also impacted the composition of the efficient frontier, as shown in S5 Text. When the attendance rates were varied, lower attendance rates were observed to shift scenarios with less restrictive criteria, especially with regards to smoking behavior, on the efficient frontier (S5 Text Tables A–C). This may be due to the fixed costs of the screening scenarios, which are independent of the number of screened individuals; at lower levels of participation these costs have a greater influence on the cost-effectiveness than the costs of CT examinations.

When the attributable costs were varied, it was observed that halving the attributable costs had little effect on the scenarios on the efficient frontier (S5 Text Table D). When the attributable costs were doubled, it was observed that scenarios with less restrictive criteria, especially with regards to smoking cessation, were shifted on the efficient frontier (S5 Text Table E).

When the costs of CT examinations were varied, it was observed that halving the costs of CT examinations also shifted scenarios with less restrictive criteria, in particular with regards to smoking cessation, on the efficient frontier (S5 Text Table F). Doubling the costs of CT examinations had the greatest effect of all sensitivity analyses; the scenarios with the most restrictive criteria with regards to age and smoking were shifted on the efficient frontier and the least costly scenarios on this efficient frontier favored biennial screening (S5 Text Table G).

Scenario #2 was on the efficient frontier across all sensitivity analyses, with the exception of assuming the lowest attendance rates (S5 Text Table H). In contrast, although Scenario #5 closely resembles the eligibility criteria that were used in the NLST, it was not on the efficient frontier in any of the sensitivity analyses.

## Discussion

This simulation study indicates that lung cancer screening can be cost-effective in a population-based setting when eligibility is restricted to high-risk groups. In contrast, utilizing loose eligibility criteria yields nonoptimal and potentially cost-ineffective scenarios, as the cost-effectiveness of lung cancer screening is highly dependent on scenario characteristics, primarily the smoking eligibility criteria. Scenarios that utilize stringent smoking eligibility criteria are more cost-effective than scenarios that utilize less restrictive smoking eligibility criteria due to a focus on individuals at higher risk of developing lung cancer. This greatly reduces the number of screening examinations while still screening those at highest risk. Thus, the level of lung cancer risk at which an individual is eligible for lung cancer screening should be considered before implementing lung cancer screening policies. Future research should investigate the cost-effectiveness of lung cancer screening selection based on accurate lung cancer risk prediction models using suitable risk thresholds [41–43].

The results of this study suggest that the greater reduction in lung cancer mortality and number of life-years gained by annual screening outweigh the costs of the additional number of CT examinations compared with biennial screening, which has previously been suggested to be equally or more cost-effective than annual screening [14,44]. However, previous studies indicated that lung cancer may be more difficult to detect in stage IA with biennial screening [24]. As survival in stage IA is considerably higher compared with other stages, the potential for mortality reduction and life-years gained is higher for annual screening compared to biennial screening [45]. This is supported by the modeling study that informed the USPSTF, which showed that annual screening provides substantial benefits over biennial screening at modest diminishing returns [13].

Previous studies that examined the cost-effectiveness of lung cancer screening only considered limited numbers of screening scenarios, which provided limited information on the effects of scenario characteristics [4–8]. The results of this study suggest that scenario characteristics, especially smoking eligibility criteria and screening interval, influence the cost-effectiveness of a scenario and suggest that a large variety of scenarios should be considered. In addition, considering a wide variety of screening scenarios provides sufficient comparator scenarios to yield appropriate ICERs [18]. Previous studies often reported the ACERs of the investigated screening scenarios as the ICERs, which can give misleading cost-effectiveness estimates [17]. This study provides both the ACERs and the ICERs of the scenarios on the efficient frontier, in contrast to previous studies that generally did not report an efficient frontier [4–8]. The robustness of the scenarios on the efficient frontier in this study is demonstrated by the sensitivity analyses of all 576 scenarios. This study incorporates both allocated costs for all screening-related procedures and attributable costs for various stages of lung cancer care, which were derived from government data in a province with universal health care, which allows for more comprehensive and accurate cost estimates compared with other studies. Furthermore, this study incorporates detailed information on smoking behavior and smoking-related mortality in contrast to previous studies. Finally, although the majority of previous studies only reported the number of life-years gained, this study reports a variety of benefits (such as lung cancer mortality reduction and the number of life-years gained) and harms (such as the number of false-positive results and the occurrence of overdiagnosis).

This study has some limitations; for example, quality of life was not incorporated in the analyses. There may be some differences in quality of life between annual and biennial screening, as more frequent screening will increase the impact of screening and follow-up–related effects on quality of life. However, results from the NELSON trial indicate that although CT lung cancer screening has a minor impact on quality of life in the short term, the long-term effects are negligible [46,47]. In addition, utility estimates for lung cancer care are highly variable [48].

Another limitation is that assumptions for follow-up procedures were based on data from the NLST, which may not be generalizable to a population-based setting, as screening algorithms with reduced false-positive rates are being investigated [1,49–51]. By reducing the false-positive rates, the number of unnecessary follow-up CTs and invasive diagnostic procedures may be reduced as well, further improving the cost-effectiveness of lung cancer screening.

Finally, although fully allocated costs for lung cancer care and observed costs for the administration of a cancer screening program were incorporated in the analyses, the government of Ontario only reimburses the physician costs of a CT examination. However, capital investments would be required to acquire the CT scanners necessary to implement a lung cancer screening program, which could influence the costs per CT examination. Conversely, the increased CT capacity could potentially lead to discounts on the costs per CT examination.

This study used a cost-effectiveness threshold of $50,000 Canadian dollars ($41,114 May 1, 2015, US dollars) per life-year gained, similar to previous Canadian cost-effectiveness studies [39]. However, the acceptable ratio between costs and effects differs between countries. For example, although a cost-effectiveness threshold of US$100,000 per quality-adjusted life-year has been proposed for the US, the United Kingdom’s National Institute for Health and Care Excellence uses a £20,000–£30,000 ($30,274–$45,411 May 1, 2015, US Dollars) threshold to determine cost-effectiveness [3,52,53]. Thus, the optimal screening scenario depends in part on the chosen cost-effectiveness threshold: if a cost-effectiveness threshold of $60,000 Canadian dollars ($49,336 May 1, 2015, US dollars) per life-year gained was chosen, Scenario #3 (annual screening for persons aged 55–75 who smoked at least 30 pack-years and currently smoke or quit smoking less than 10 y ago) would have been considered the optimal scenario. However, the ICER of Scenario #2 (annual screening for persons aged 55–75 who smoked at least 40 pack-years and currently smoke or quit smoking less than 10 y ago) remained below the proposed cost-effectiveness threshold of $50,000 Canadian dollars per life-year gained in 5 out of 7 sensitivity analyses (71.4%) with a range of $28,630–$73,507 Canadian dollars per life-year gained. This suggests that both the dominance and cost-effectiveness of Scenario #2 are robust across various sensitivity analyses.

Although our results suggest that a uniform biennial screening interval is dominated by a uniform annual screening interval, recent studies suggest it may be possible to identify individuals for whom biennial screening intervals could be recommended. NLST participants with a negative prevalence screen had a substantially lower risk of developing lung cancer compared to individuals with a positive prevalence screen [54]. Results from the NELSON trial suggest that the 2-y probability of developing lung cancer after a CT screen varied substantially by nodule size and volume doubling time [51]. Future research should evaluate whether the interval between screens can be varied based on previous screening results and what impact this has on cost-effectiveness. In addition, precision medicine could improve the treatment of selected individuals, and biomarkers might help to distinguish between indolent nodules and aggressive nodules requiring rapid diagnosis and treatment. The impacts of these future developments need to be assessed.

In conclusion, this study indicates that lung cancer screening can be cost-effective in a population-based setting if stringent smoking eligibility criteria are applied. Annual screening scenarios are more cost-effective than biennial screening scenarios.

## Supporting Information

S1 Appendix Supplemental Figures A–H.
(DOCX)

S1 Text MISCAN-Lung model structure. (DOCX)

S2 Text Smoking behavior and smoking-related mortality. (DOCX)

S3 Text Lung cancer incidence in Ontario. (DOCX)

S4 Text Screening-related follow-up procedures. (DOCX)

S5 Text Impact of sensitivity analyses. (DOCX)

S6 Text Data Availability Statement. (DOCX)

## Abbreviations

ACER: average cost-effectiveness ratio
CT: computed tomography
ICER: incremental cost-effectiveness ratio
NELSON: Dutch–Belgian lung cancer screening trial
NLST: National Lung Screening Trial
USPSTF: United States Preventive Services Task Force
