# Supplementary material for: Performance and Cost-Effectiveness of Computed Tomography Lung Cancer Screening Scenarios in a Population-Based Setting: A Microsimulation Modeling Analysis in Ontario, Canada
Source: PLoS Med. 2017 Feb 7;14(2):e1002225. doi: 10.1371/journal.pmed.1002225 (PMC5295664; doi:10.1371/journal.pmed.1002225)
Supplement: S1 Appendix — Supplemental Figures A–H. (DOCX) [file pmed.1002225.s001.docx]

**S1 Appendix: Supplemental Figures A-H**

**Supplemental Figure A: Cost-effectiveness of lung cancer screening scenarios by screening starting age in the base-case analysis**

**
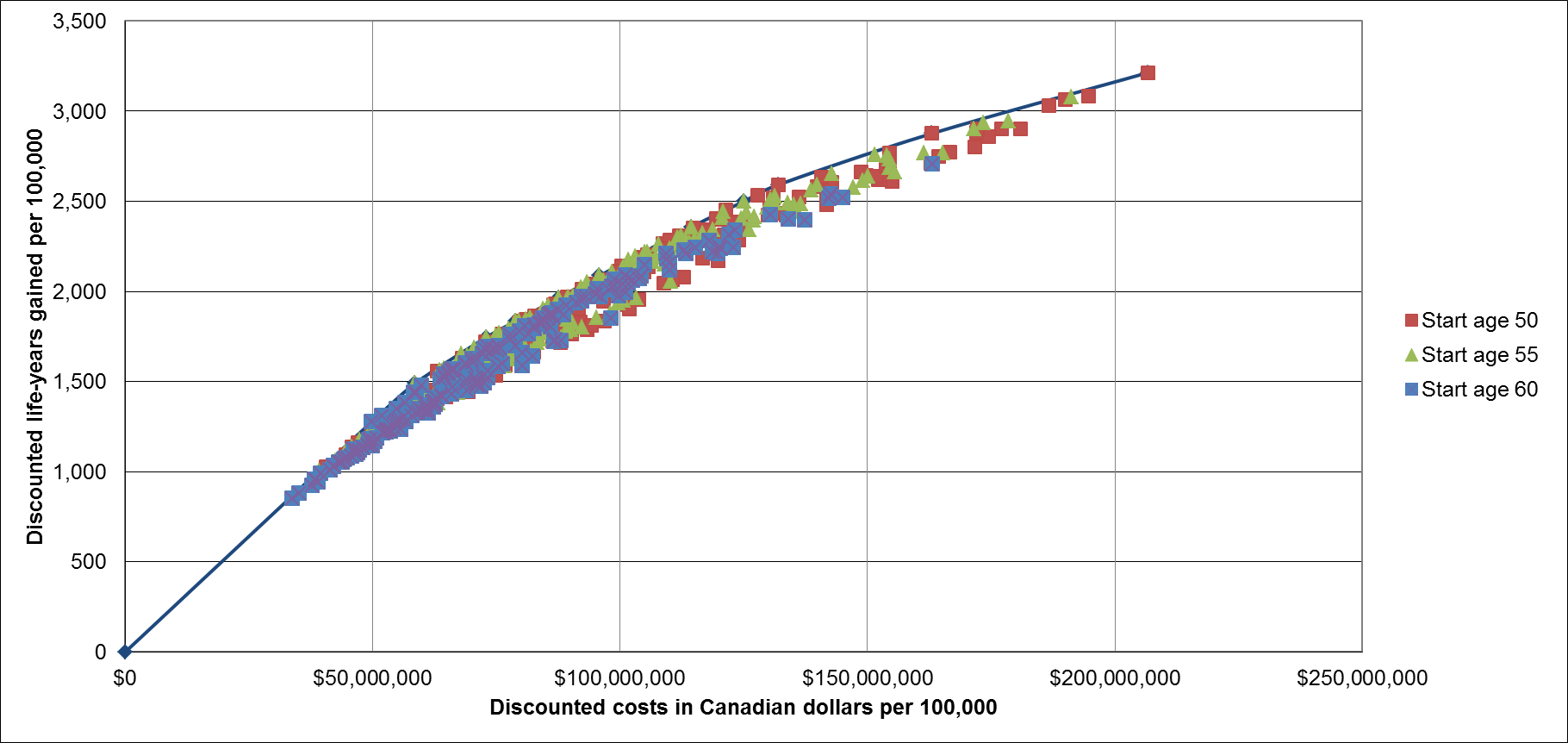
**

**Supplemental Figure B: Cost-effectiveness of lung cancer screening scenarios by screening stopping age in the base-case analysis**

**
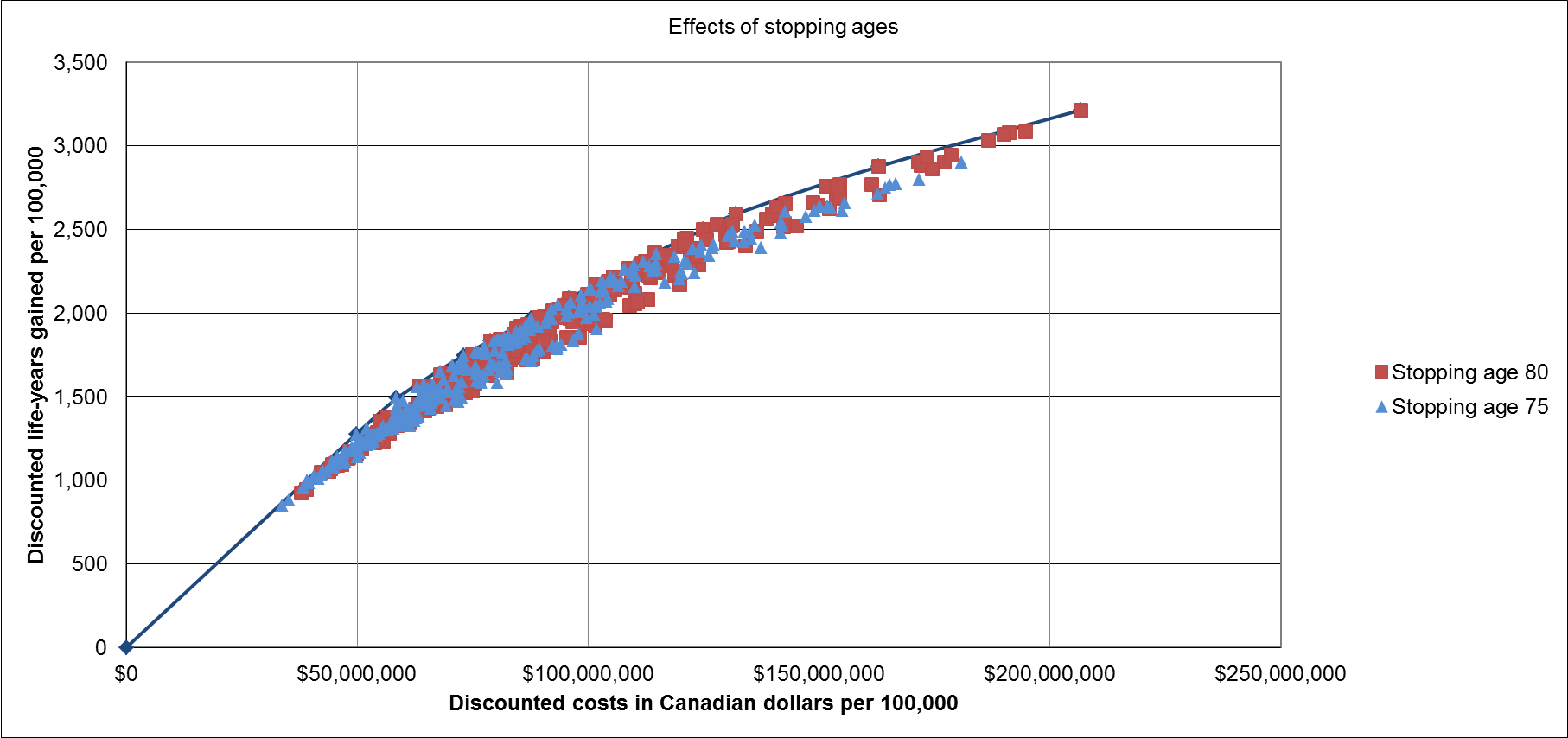
**

**Supplemental Figure C: Cost-effectiveness of lung cancer screening scenarios by type of aggregated smoking criteria in the base-case analysis**

**
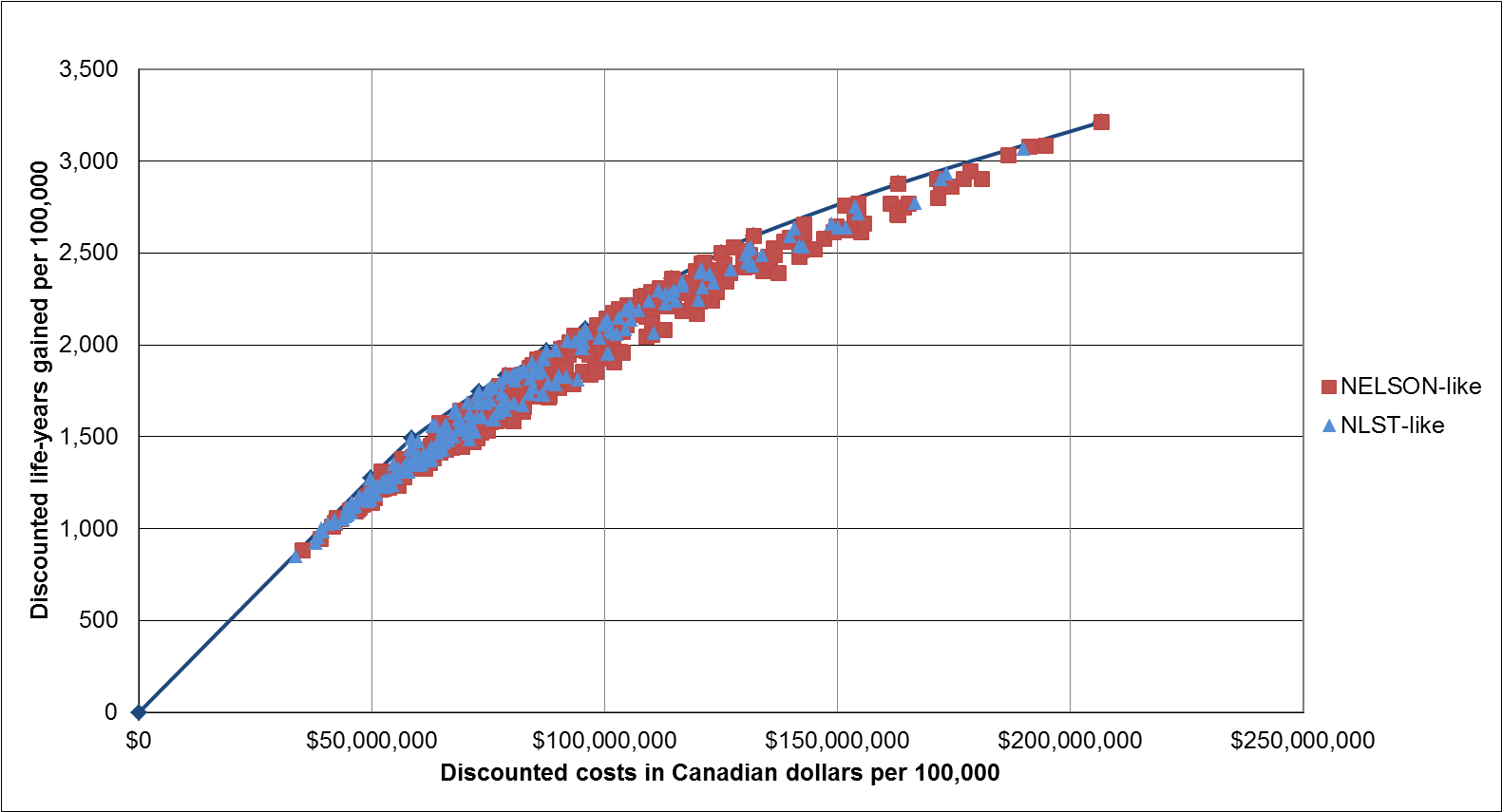
**

**Supplemental Figure D: Cost-effectiveness of lung cancer screening scenarios by aggregated smoking criteria (efficient frontier and NLST-like scenarios only) in the base-case analysis**

**
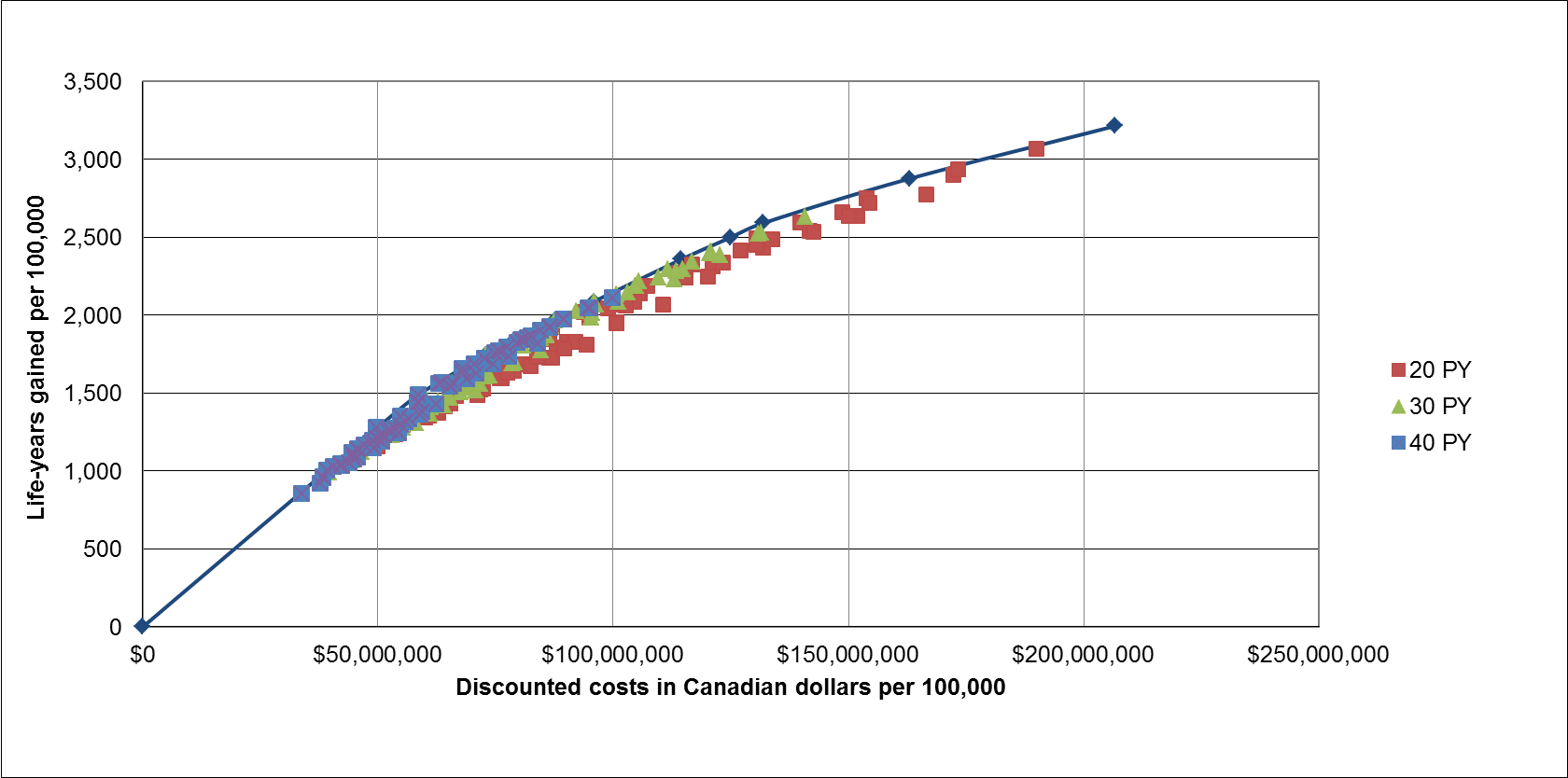
**

**Figure notes:** abbreviations: pack-years (PY)

**Supplemental Figure E: Cost-effectiveness of lung cancer screening scenarios by aggregated smoking criteria (efficient frontier and NELSON-like scenarios only) in the base-case analysis**

**
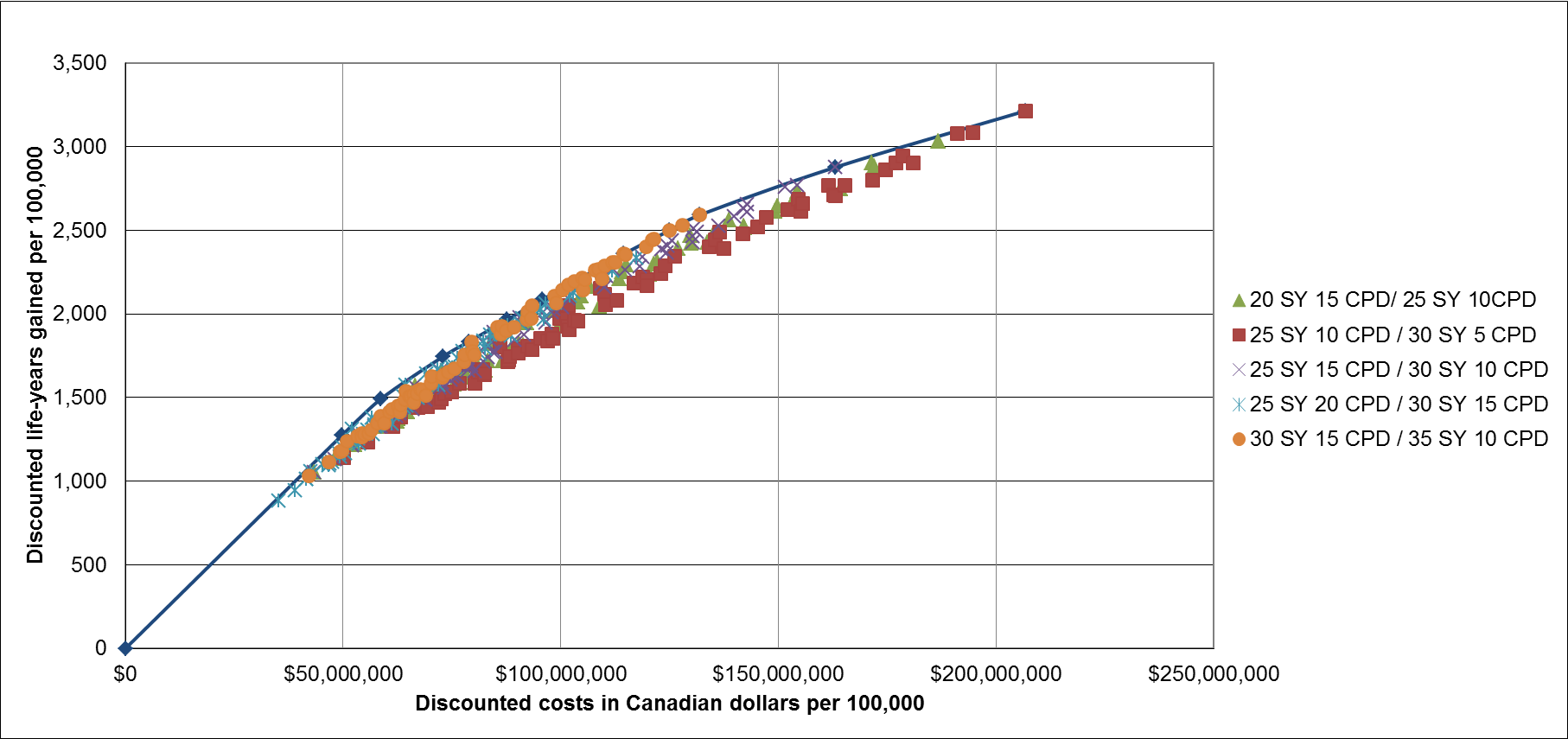
**

**Figure notes:** abbreviations: smoking duration in years (SY), cigarettes per day (CPD)

**Supplemental Figure F: Cost-effectiveness of lung cancer screening scenarios by years since cessation in the base-case analysis**

**
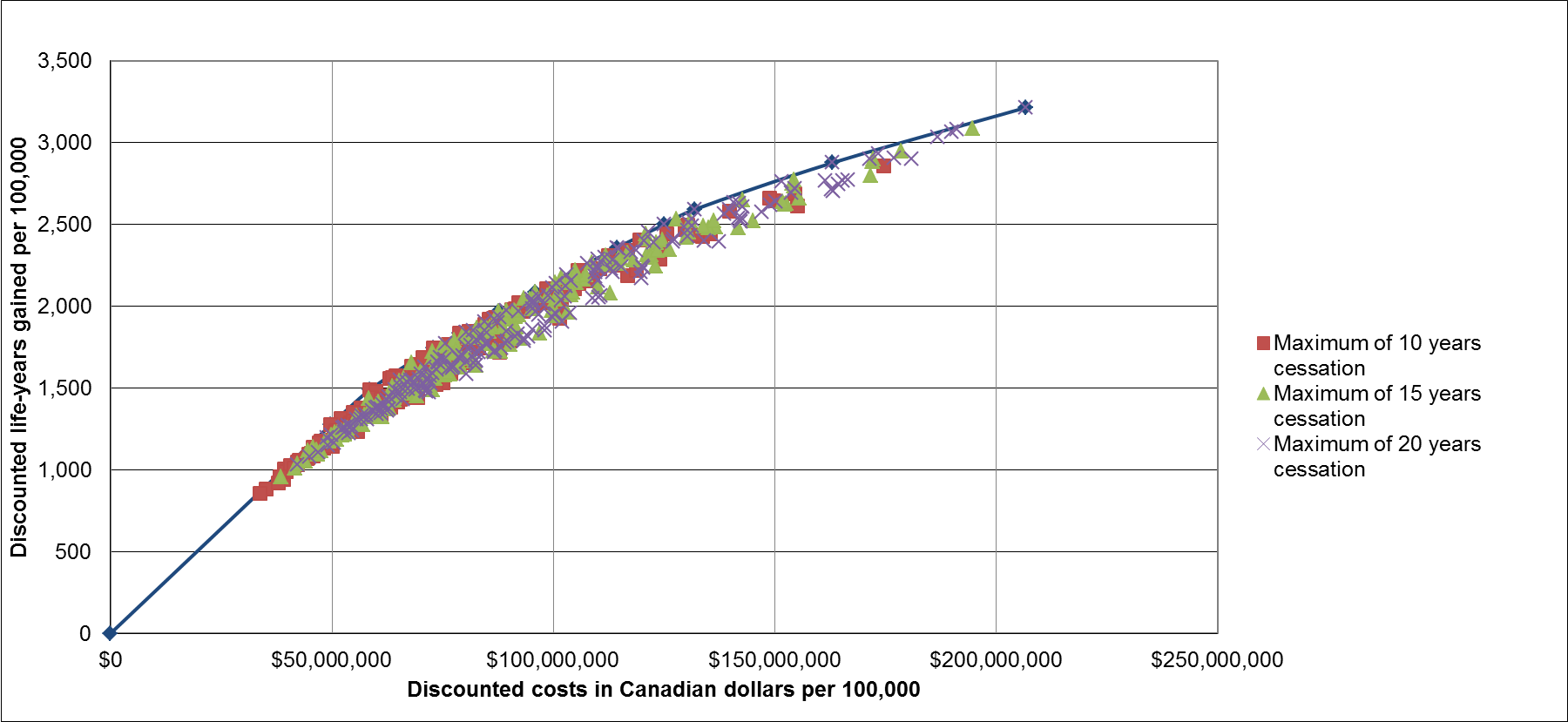
**

**Supplemental Figure G: Cost-effectiveness of lung cancer screening scenarios: exclusion of individuals after reaching the maximum number of years since cessation compared with no exclusion in the base-case analysis**

**
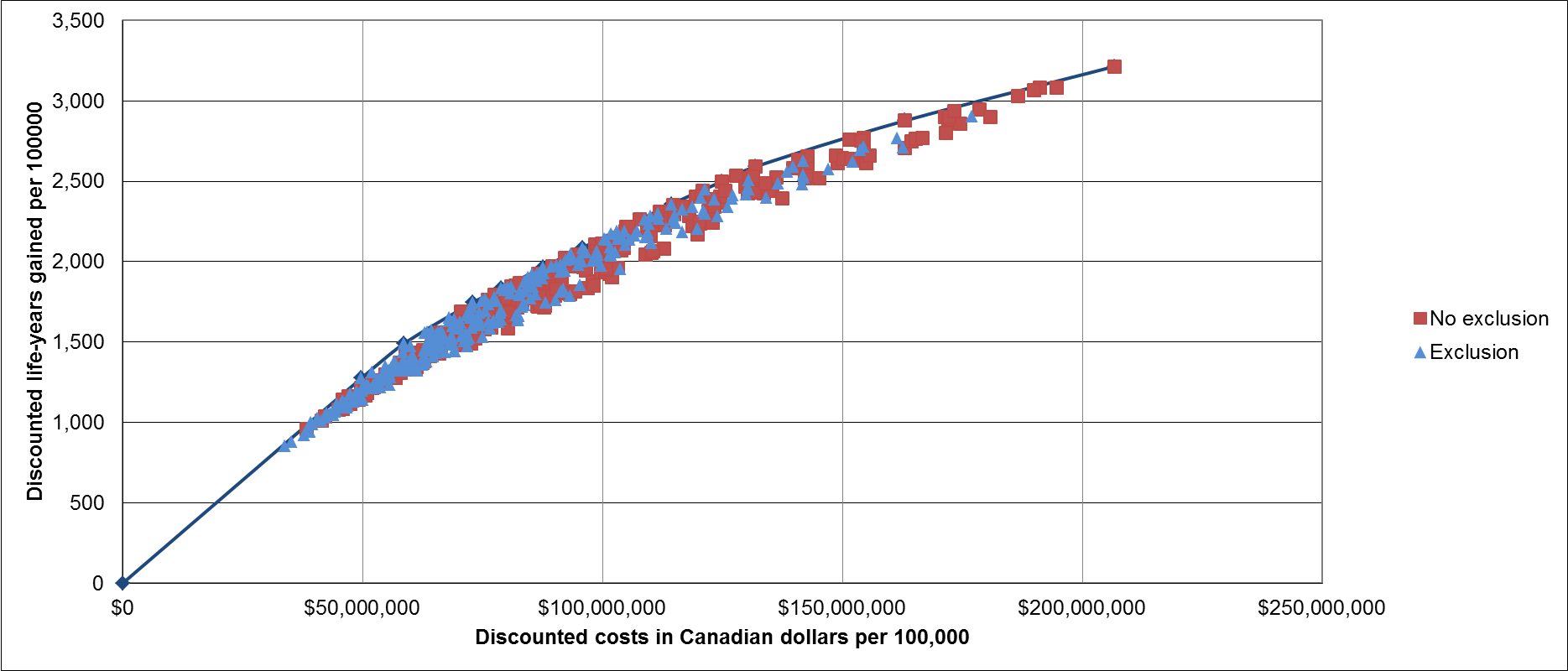
**

**Supplemental Figure H: Cost-effectiveness of lung cancer screening scenarios by intervals between screenings in the base-case analysis**

**
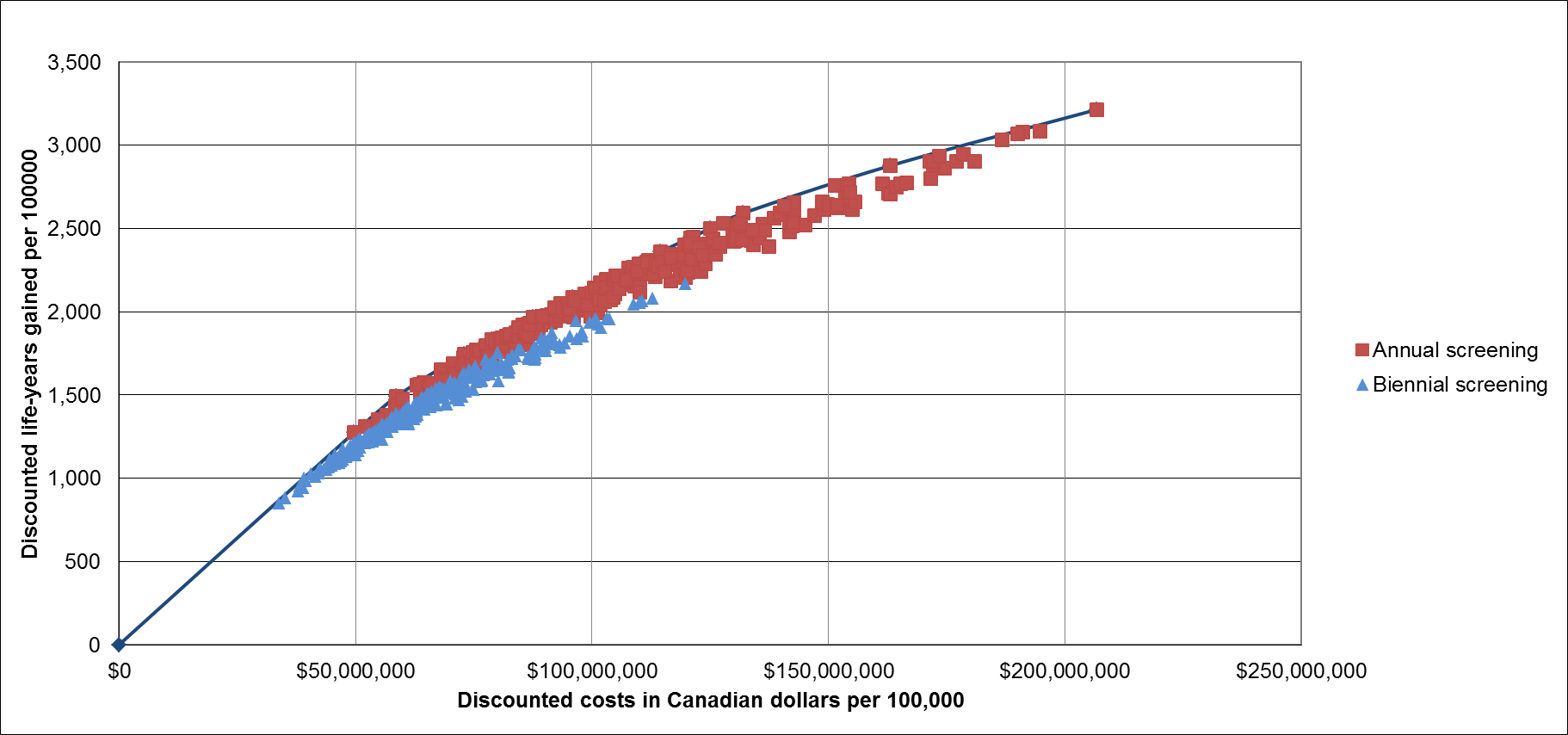
**
