# Supplementary material for: Performance and Cost-Effectiveness of Computed Tomography Lung Cancer Screening Scenarios in a Population-Based Setting: A Microsimulation Modeling Analysis in Ontario, Canada
Source: PLoS Med. 2017 Feb 7;14(2):e1002225. doi: 10.1371/journal.pmed.1002225 (PMC5295664; doi:10.1371/journal.pmed.1002225)

**Supplement S1: MISCAN-Lung model structure**

The MIcrosimulation SCreening ANalysis (MISCAN) Lung model simulates life histories for each individual in the considered population from birth until death, in the presence and absence of screening. Through comparing the life histories in the presence of screening with the corresponding life histories in the absence of screening, MISCAN-Lung can quantify the effectiveness of a screening scenario and the accompanying costs. The following sections detail the model structure of the MISCAN-Lung model.

**General model structure**

The MISCAN-Lung model is a stochastic, microsimulation model programmed in Delphi (Borland Software Corporation, Scotts Valley, California, United States). MISCAN-Lung was developed for the evaluation of screening for lung cancer and to investigate the effects of tobacco-control policies. MISCAN-Lung is a semi-Markov model, which generates durations for each state. Individuals are simulated one at a time, which allows future state transitions to depend on past transitions giving the model a “memory”. MISCAN-Lung simulates sequences of events by drawing from distributions of probabilities/durations, which makes the results of the model subject to random variation.

MISCAN-Lung consists of several modules: a demography/smoking history generator module, a smoking-dose response module for lung carcinogenesis, a natural history module and a screening module.

**Demography/smoking history generator module**

First, birth-tables, representative for the population under consideration, are used to draw a date of birth for each simulated individual. Age, gender and cohort specific smoking initiation probabilities, representative for the population under consideration, are used to determine whether an individual initiates smoking and the age of smoking initiation. Upon smoking initiation, persons enter one of five smoking intensity categories. Age, gender, cohort and smoking intensity category specific average number of cigarettes smoked per day are generated for each individual that initiates smoking. If an individual initiates smoking, age, gender and cohort specific smoking cessation probabilities are used to determine whether an individual ceases smoking and the age of smoking cessation. Details on the modeling of smoking behavior and smoking related mortality are presented in Supplement S2: Smoking behavior and smoking related mortality.

**Smoking related mortality**

Upon generating a person’s smoking history, the age of death from causes other than lung cancer is generated, using mortality probabilities based on the person’s smoking history (smoking duration, smoking intensity category and average number of cigarettes per day, smoking status and years since cessation, if applicable), year of birth, age and gender. The maximum age an individual can achieve in MISCAN-Lung is exactly 100 years.

**Smoking-dose response module for lung carcinogenesis**

The smoking-dose response module is used to model lung carcinogenesis as a function of a person’s age, gender and smoking history. MISCAN-lung utilizes the two-stage clonal expansion model (TSCE) as described by Heidenreich et al., as its smoking-dose response module [1]. The parameters of the TSCE were obtained through calibration to the Nurses’ Health Study and the Health Professionals Follow-up Study [2]. However, the gender-specific parameters for malignant transformation were recalibrated to data from the National Lung Screening Trial (NLST), the Prostate, Lung, Colorectal and Ovarian cancer screening trial (PLCO) and the Surveillance, Epidemiology, and End Results (SEER) Program [3].

**Natural history module**

**Transitions**

Lung cancers are assumed to progress sequentially through stages IA to IV, as shown in Figure A. The probability that a lung cancer progresses to a more advanced preclinical stage or is diagnosed clinically (e.g. diagnosed due to symptoms) is modeled by histology and stage. After clinical diagnosis, lung cancer survival is simulated using gender-, stage-, and histology specific survival estimates, obtained from SEER-17 [4]. The date of death for individuals with lung cancer is set to the earliest simulated date of death (either due to lung cancer or other causes).

**Transition probabilities and durations in states**

The preclinical durations (in the absence of screening), by histology, stage and gender were calibrated to the rates of screen‐detected and interval cancers observed in the NLST and PLCO trials using individual-level data [3]. The preclinical durations (in the absence of screening) are drawn from Weibull distributions. The transition probabilities and correlations between durations of preclinical cancer stages have been described previously [3].

**Screening module**

Screening may detect cancers in each of the preclinical screen-detectable states, depending on the sensitivity of the screening test for the specific histology and preclinical stage. The model parameters for CT sensitivity by preclinical stage and histology and the effectiveness of CT screening were calibrated to individual-level data from the NLST [3]. Upon detection of lung cancer by screening, a person’s life history may be altered. Detection by screening may cure a patient, allowing him to resume his normal (lung cancer free) life history. The probability of cure differs by the stage at detection. Negative effects of screening, such as overdiagnosis of lung cancer (described subsequently), are also modeled.

**Integrating modules**

Figure B shows an example of how the model integrates the different modules to determine the benefits of screening. The demography/smoking history generator module first generates a date of birth, smoking history and date of death from causes other than lung cancer. This creates a life-history in the absence of lung cancer for Person 1 (shown in life history 1). The smoking-dose response module uses the simulated smoking history to determine whether and when lung carcinogenesis occurs for Person 1 (shown in life history 2). After lung carcinogenesis occurs, the natural history model generates the progression of the cancer, which is diagnosed because of symptoms in stage II and results in a death due to lung cancer, before the death due to causes other than lung cancer would have occurred (shown in life history 1). In the screening module, a screening examination is simulated, as indicated by the arrow (shown in life history 3). The cancer is detected at the examination and, in this case, the earlier detection allows for successful treatment of the cancer. As a result, the lung cancer death is prevented and the person’s life is prolonged.

Screening may also cause harms, as shown for Patient 2 in Figure C. In Patient 2 lung cancer also develops, but the cancer would not have been clinically detected without screening (shown in life history 2). However, the cancer is screen-detected in stage IA during the screening examination simulated in the screening module (shown in life history 3). Thus, in this patient, screening detects a lung cancer that would have never been diagnosed if screening had not occurred, resulting in an overdiagnosed case. Thus, for Patient 2 screening does not provide any benefits, but results in life-years with lung cancer care that would not have occurred otherwise (overtreatment).

**References**

1. Heidenreich WF, Luebeck EG, Moolgavkar SH. Some Properties of the Hazard Function of the Two-Mutation Clonal Expansion Model. Risk Anal. 1997;17(3):391-9. doi: 10.1111/j.1539-6924.1997.tb00878.x.

2. Meza R, Hazelton W, Colditz G, Moolgavkar S. Analysis of lung cancer incidence in the nurses’ health and the health professionals’ follow-up studies using a multistage carcinogenesis model. Cancer Causes Control. 2008;19(3):317-28. doi: 10.1007/s10552-007-9094-5.

3. ten Haaf K, van Rosmalen J, de Koning HJ. Lung Cancer Detectability by Test, Histology, Stage, and Gender: Estimates from the NLST and the PLCO Trials. Cancer Epidemiology Biomarkers & Prevention. 2015;24(1):154-61. doi: 10.1158/1055-9965.epi-14-0745.

4. Surveillance, Epidemiology, and End Results (SEER) Program ([www.seer.cancer.gov](http://www.seer.cancer.gov)) SEER*Stat Database: Incidence - SEER 18 Regs Research Data + Hurricane Katrina Impacted Louisiana Cases, Nov 2012 Sub (1973-2010 varying) - Linked To County Attributes - Total U.S., 1969-2011 Counties, National Cancer Institute, DCCPS, Surveillance Research Program, Surveillance Systems Branch, released April 2013, based on the November 2012 submission.. Accessed March, 29 2014.

**Figure A: Lung cancer progression in the MISCAN-Lung model**


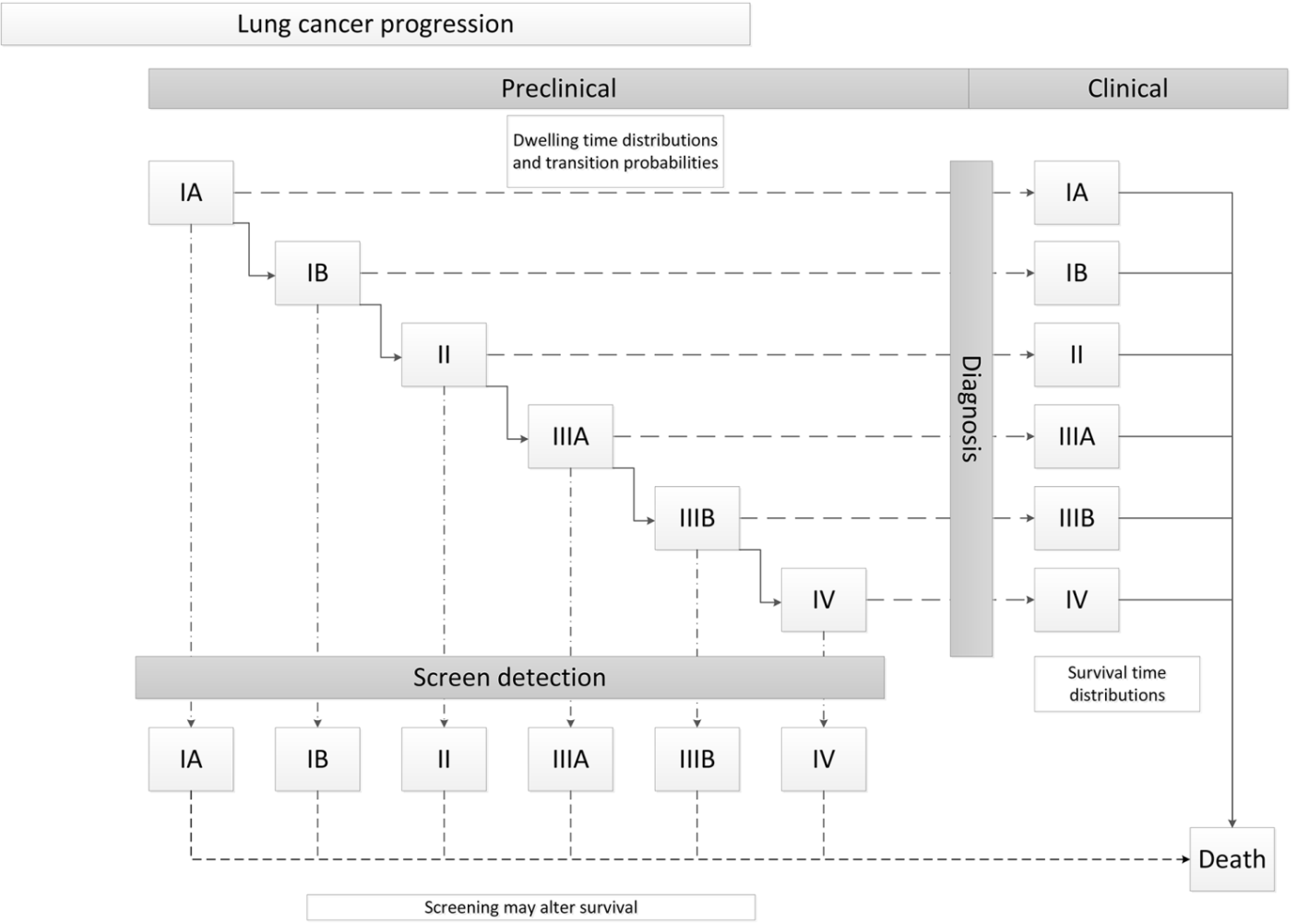


**Figure notes:** Once lung cancer has developed, it will progress from less advanced to more advanced preclinical stages until it is clinically detected. This process is similar for all histologies, however, the average time spent in the current state differs by histology, preclinical cancer stage and gender. The probability that a cancer progresses to a more advanced preclinical stage or is diagnosed clinically (e.g. diagnosed due to symptoms) is modeled by histology and stage. Screening may detect cancers in each of the preclinical screen-detectable states, depending on the sensitivity of the screening test for the specific histology and preclinical detectable state. Upon detection of lung cancer by screening, a person’s life history may be altered. Detection by screening may cure a patient, allowing him to resume his normal (lung cancer free) life history. The probability of cure differs by the stage of detection. After clinical detection or screen detection (without cure) the patient’s duration of survival follows a histology and stage specific survival function, which is piecewise uniformly distributed. A person may also die from causes other than lung cancer.

**Figure B: Integrating modules: modeling benefits of screening**
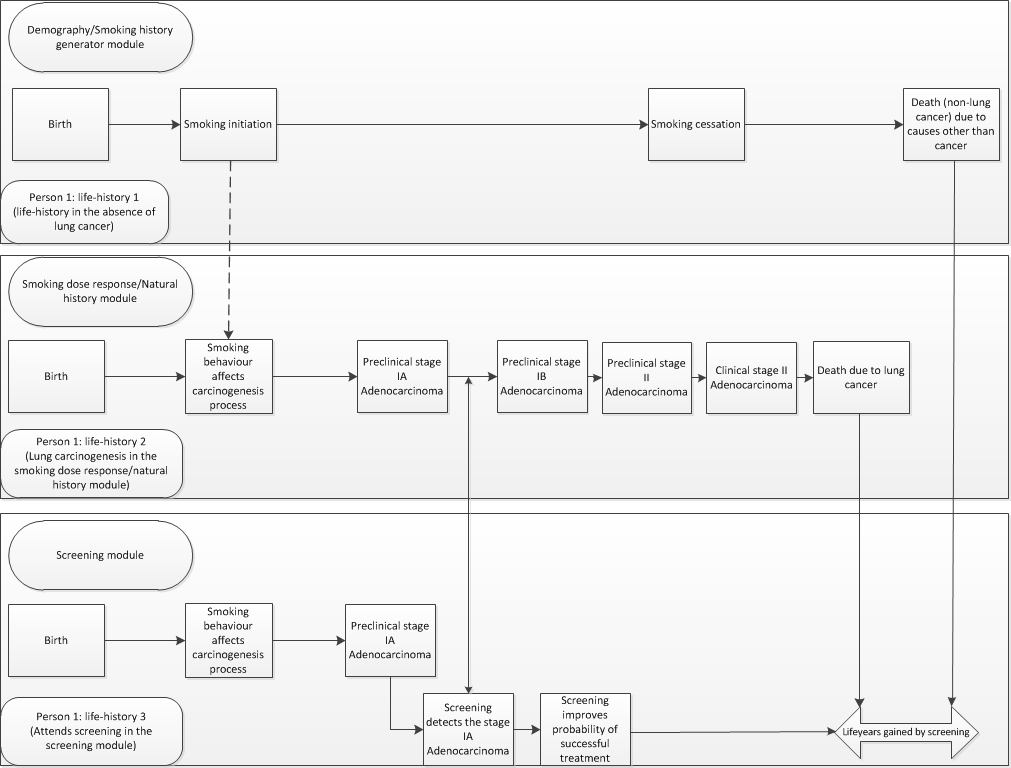


**Figure C: Integrating modules: modeling harms of screening**


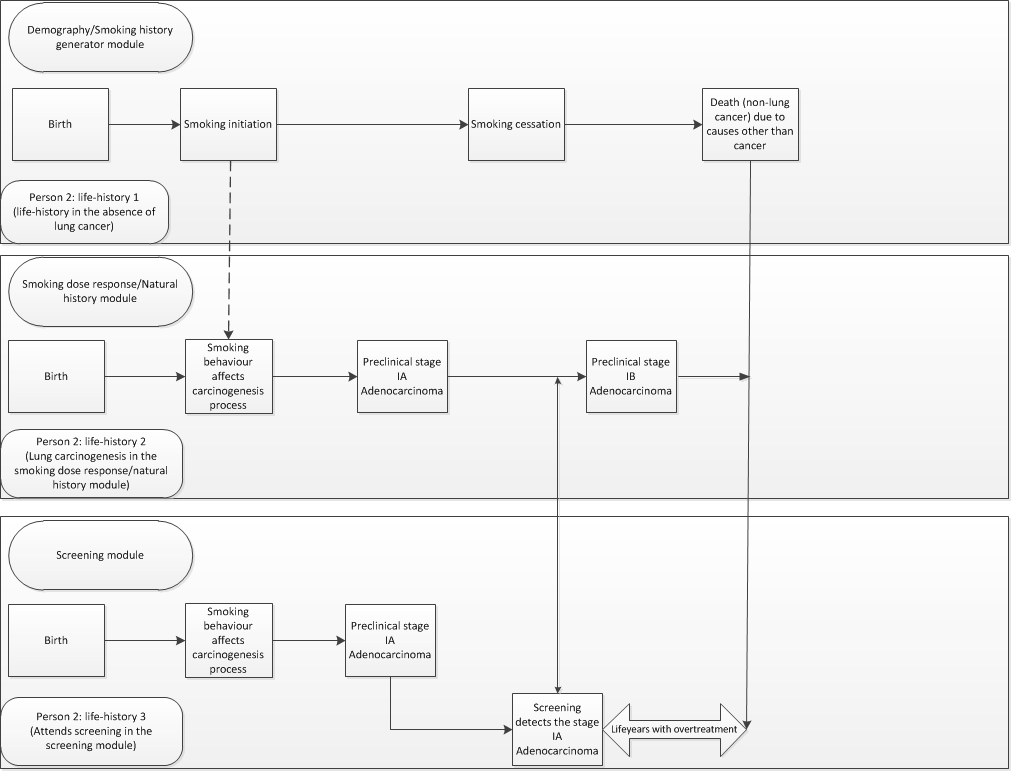

Supplement: S1 Text — (DOCX) [file pmed.1002225.s002.docx]
