# Supplementary material for: Performance and Cost-Effectiveness of Computed Tomography Lung Cancer Screening Scenarios in a Population-Based Setting: A Microsimulation Modeling Analysis in Ontario, Canada
Source: PLoS Med. 2017 Feb 7;14(2):e1002225. doi: 10.1371/journal.pmed.1002225 (PMC5295664; doi:10.1371/journal.pmed.1002225)
Supplement: S2 Text — (DOCX) [file pmed.1002225.s003.docx]

**Supplement S2: Smoking behavior and smoking related mortality**

The MISCAN-Lung model incorporates detailed information on smoking behavior in Ontario, such as smoking initiation and cessation probabilities and the average number of cigarettes smoked per day (divided into five categories) by cohort, age and gender. In addition, the effects of smoking on non-lung cancer mortality are incorporated. The following sections detail the methods and assumptions used to generate the smoking behaviors of individuals and the effects of smoking on non-lung cancer mortality in the MISCAN-Lung model.

**Smoking initiation**

Little data are available on Ontario-specific smoking initiation by age. Previous investigations in the U.S. indicate that the majority of smoking initiation occurs before the age of 30 for cohorts born after the early 1900’s [1,2]. Anderson et al. have shown that age-specific smoking initiation probabilities (the probability that a never-smoker, a person that smoked less than 100 cigarettes during his or her life-time, at the beginning of that year of age starts smoking) can be estimated by calibrating them to the ever-smoking prevalence at age 30 (when the majority of initiation has occurred) [2]. Therefore, Ontario-specific data on the ever-smoking prevalence at age 30 for each investigated birth cohort (persons born between 1940-1949, 1950-1959 and 1960-1969) was obtained from the Canadian Community Health Surveys (CCHS), the National Population Health Surveys (NPHS), the Canada Health Survey, the General Social Surveys (years 1985 and 1991) and the Smoking Habits of Canadians Surveys [3-8].

For each cohort, age and gender specific smoking initiation probabilities were estimated in R package, using exponential functions, to match the observed ever-smoking prevalence at age 30 [9]. The smoking initiation probabilities were corrected for all-cause mortality using Ontario specific all-cause mortality life tables by birth-year, age and gender, obtained from the Canadian Human Mortality Database (CHMD) [10]. It was assumed persons in each cohort can initiate smoking from ages 8 to 29. Furthermore, it was assumed that the age-specific smoking initiation probabilities increase with age until age 17, after which the probability of smoking initiation decreases, as shown in previous investigations [1]. Table A shows the observed ever-smoking prevalence at age 30 compared to the estimated ever-smoking prevalence at age 30 for each cohort, by gender. Overall, the estimated prevalences at age 30 closely match the observed data.

**Smoking cessation probabilities**

Information on Ontario-specific current-, former- and never-smoker prevalences for each cohort, by gender, was obtained from the CCHS, the NPHS, Canada Health Survey, General Social Surveys (years 1985 and 1991) and the Smoking Habits of Canadians Surveys [3-8]. Smokers who attempt to quit have a high probability to relapse in their first two years since cessation, therefore, we defined former smokers as smokers who reported having quit for at least two years [2]. However, the available surveys did not inquire about the time since cessation until 2000 and surveys held after 2000 had limited sample sizes. Therefore, for each cohort, information on current-, former- and never-smoking prevalences in the year 2000 were used as calibration targets to estimate the age-specific smoking cessation probabilities (the probability that a current smoker at the beginning of that year of age ceases smoking permanently) for each cohort, by gender.

It was assumed that current smokers can cease smoking from age 9 to 100 (the maximum age in MISCAN-Lung) onwards. The probability of successful smoking cessation is assumed to increase with age until age 85, after which the probability of smoking cessation is assumed to be similar to that at age 85 [1,11,12]. The smoking cessation probabilities were estimated in R package by age, gender and cohort, using logistic functions [9]. The smoking cessation probabilities were estimated simultaneously with the mortality probabilities by smoking behavior (detailed in the “Mortality by smoking behavior” section of this Supplement).

Table B shows the observed current-, former- and never-smoking prevalence in 2000 compared to the estimated prevalence for each cohort, by gender. Overall, the estimated probabilities reproduce the current-, former- and never-smoking prevalences in 2000.

**Cigarettes Smoked per Day**

Data on the average number of cigarettes smoked per day (CPD) were obtained from the CCHS, the NPHS, Canada Health Survey, General Social Surveys (years 1985 and 1991) and the Smoking Habits of Canadians Surveys [3-8]. Smokers were divided into five smoking-intensity quintiles, ranging from the lightest to heaviest smokers by the reported average number of CPD at each age, similar to Anderson et al. [2]. Age-specific values for the average number of CPD per quintile for ages ≥30 were calculated for ages 30, 40, 50, 60 and 70, depending on the availability of data for each cohort. To account for underreporting of the number of cigarettes per day, due to digit preference and the social undesirability of smoking, the observed values were increased by 7.5%, as this provided the best fit to the observed overall mortality and lung cancer incidence in Ontario (see the following section “Mortality by smoking behavior” and Supplement S3: Lung cancer incidence in Ontario) [13,14]. Linear interpolation was used to fit the average number of CPD by age and quintile between observed CPD values. CPD values were extrapolated by assuming the average CPD value in each quintile decreases by 1% yearly, to reflect the reduction in CPD smoked as individuals age beyond the age of 45-50 [15]. Figures A and B illustrate the variation in average CPD across the five quintiles in the 1940-1949 cohort, for men and women respectively.

In MISCAN-Lung, upon smoking initiation, an individual is randomly assigned to a quintile (with equal probabilities for each quintile) in which the individual will remain until smoking cessation or death. Similar to Anderson et al., smoking behavior was divided into a period of smoking uptake (ages under age 30) and smoking maintenance after the age of 30 [2]. The number of CPD for persons younger than 30 are modeled by applying the uptake formulas described by Anderson et al., as implemented in the U.S. Smoking History Generator, to the average number of cigarettes per day at age 30 for that person’s smoking quintile [2,16]:

$$UptakeMale\left( currentsmokingduration,calenderyear,currentage \right)=-38.578+3.342*\sqrt{currentsmokingduration}-0.00168*{\max\left( 79,calenderyear-1900 \right)}^{2}-17.538*\sqrt{currentage}+44.967*ln(currentage)$$

$$UptakeFemale\left( currentsmokingduration,calenderyear,currentage \right)=-56.751+0.700*currentsmokingduration-0.00163*{\max\left( 79,calenderyear-1900 \right)}^{2}-3.473*currentage+32.8*\sqrt{currentage}$$

The uptake formulas are scaled so that the number of CPD the person smokes at age 30 matches that of the average number of CPD in the quintile the person belongs to, regardless of the age of initiation.

**Mortality by smoking behavior**

Ontario specific all-cause mortality life tables by birth-year, age and gender, were obtained from the CHMD [10]. To account for competing risks, these life tables were corrected for lung cancer mortality through subtracting the probability of dying from lung cancer from the probability of dying from all causes for each age, as described by Rosenberg et al. [17].

Information on lung cancer mortality by five-year age groups and gender for years 1990-2009 was obtained from the Ontario Cancer Registry through an electronic copy located on a secure server at the Institute for Clinical Evaluative Sciences [18]. For each available year, the probability of dying from lung cancer was calculated by age group and gender.

The registry data indicated that dying from lung cancer rarely occurs before ages 20-24. Therefore, we assumed the probability of dying from lung cancer to be zero for ages 0-19 for all cohorts. For cohorts with missing data for age-groups before 1990, we assumed that the probability of dying from lung cancer in those age-groups in the years before 1990 was similar to that of their respective age-groups in 1990. Similarly, the probabilities of dying from lung cancer for age-groups in the years after 2009 were assumed to be similar to those of the respective age groups in 2009.

The life tables corrected for lung cancer mortality were then further corrected for smoking behavior, for each birth-year and gender. First, it was assumed that smoking behavior influences non-lung cancer mortality from age 40 onwards, similar to Rosenberg et al. [17]. Thus, before age 40, the non-lung cancer mortality probabilities for never -and ever-smokers with the same birth-year are assumed to be similar:

$$P\left( nonlungcancermortality_{neversmoker}, currentage,gender,birthyear \right)=P\left( nonlungcancermortality_{eversmoker, CPDcategory}, currentage, gender,birthyear \right)=P\left( nonlungcancermortality_{overallpopulation}, currentage, gender,birthyear \right)$$

From age 40 onward, the non-lung cancer mortality probabilities for never-smokers were assumed to be lower than those of the overall population with the same birth-year, as the overall population includes ever-smokers who have higher non-lung cancer mortality probabilities compared with never-smokers [17,19]. Therefore, after age 40, the non-lung cancer mortality probabilities of never-smokers were assumed to be similar to the non-lung cancer mortality probabilities of the overall population with the same birth-year, corrected for never-smoking:

$P\left( nonlungcancermortality_{neversmoker}, currentage,gender,birthyear \right)=P\left( nonlungcancermortality_{overallpopulation}, currentage, gender,birthyear \right)*Correctionfactor_{neversmoker}$

However, as ever-smokers have higher non-lung cancer mortality probabilities compared with never-smokers, the proportion of ever-smokers in the overall population with the same birth-year is expected to decrease at higher ages. Thus, at higher ages, never-smokers will represent a higher proportion of the overall population. As a result, the non-lung cancer mortality probabilities for never-smokers will converge to those of the overall population of that birth-year at higher ages. This convergence is assumed to start from age 70 onward and therefore, the non-lung cancer mortality probabilities of never-smokers from that age onward were assumed to be:

$P\left( nonlungcancermortality_{neversmoker}, currentage,gender,birthyear \right)=P\left( nonlungcancermortality_{overallpopulation}, currentage, gender,birthyear \right)*(Correctionfactor_{neversmoker}+\left( currentage-69*\left( \frac{\left( 1-Correctionfactor_{neversmoker} \right)}{30} \right) \right))$

As indicated previously, ever-smokers have higher non-lung cancer mortality probabilities compared with never-smokers [17,19]. However, the non-lung cancer mortality probabilities for ever-smokers are also influenced by the average number of CPD smoked by a person [17,19]. Therefore, it was assumed that non-lung cancer mortality probabilities increased with higher average numbers of CPD. Four categories of average numbers of CPD were defined, similar to Thun et al.: < 10 CPD, 10-19 CPD, 20-39 CPD and ≥ 40 CPD [19]. Furthermore, longer durations of smoking have been indicated to increase non-lung cancer mortality probabilities [19]. Therefore, the increase in non-lung cancer mortality probabilities for current-smokers compared with never-smokers was also assumed to increase with age (as a substitute for smoking duration). In addition, this increase was assumed to differ by smoking quintile, to reflect differences in the average CPD over longer periods of time. Thus, the non-lung cancer mortality probabilities for current-smokers from age 40 onward were assumed to be:

$$P\left( nonlungcancermortality_{currentsmoker_{CPDcategory,CPDquintile}}, currentage,gender,birthyear \right)=P\left( nonlungcancermortality_{neversmoker}, currentage, gender,birthyear \right)+smokingmortalityincreas{e(age)}_{CPDcategory, CPDquintile}$$

Where

$$smokingmortalityincreas{e\left( age \right)}_{CPDcategory,CPDquintile}= smokingmortalityincrease_{CPDcategory,CPDquintile}*\left( Smokingagecorrection_{CPDquintile}+((Currentage-40)*\left( \frac{\left( 1-Smokingagecorrection_{CPDquintile} \right)}{59} \right)) \right)$$

Previous research indicates that the age of smoking cessation and years since smoking cessation influence the excess risk of mortality due to past smoking behavior [17,19]. Overall, the excess risk of mortality decreases for a younger age of smoking cessation and a higher number of years since smoking cessation [17,19]. Therefore, the excess risk of non-lung cancer mortality was assumed to decrease over time for former smokers, similarly to the formula described by Rosenberg et al. [17]:

$$P\left( nonlungcancermortality_{formersmoker_{CPDcategory}}, currentage,gender,birthyear \right)=P\left( nonlungcancermortality_{neversmoker}, currentage, gender,birthyear \right)+((mortalityincreas{e(age)}_{CPDcategory,CPDquintile)}*\exp\left( \left( -0.1711+\left( 0.00102*averageCPDoverlifetime \right)+\left( 0.00171*QuitAge \right) \right)*{YearsQuit}^{1.08} \right))$$

The non-lung cancer mortality probabilities for never- and ever-smokers were estimated simultaneously with the smoking cessation probabilities to match the life tables previously corrected for lung cancer mortality for each birth-year and gender, using R package [9]. Figures C and D show the estimated all-cause mortality probabilities of the overall population for men and women born in 1955 compared to those of the CHMD life tables as examples. Overall, the estimated all-cause mortality probabilities match those of the CHMD lifetables. Figures E and F show the estimated cumulative mortality probabilities of dying from causes other than lung cancer (up to age 85) for never-smokers and current smokers (by smoking quintile) for men and women born in 1955 as examples.

**References**

1. Holford TR, Levy DT, McKay LA, Clarke L, Racine B, Meza R, et al. Patterns of Birth Cohort–Specific Smoking Histories, 1965–2009. American Journal of Preventive Medicine. 2014;46(2):e31-e7. doi: <http://dx.doi.org/10.1016/j.amepre.2013.10.022>.

2. Anderson CM, Burns DM, Dodd KW, Feuer EJ. Chapter 2: Birth-Cohort-Specific Estimates of Smoking Behaviors for the U.S. Population. Risk Analysis. 2012;32:S14-S24. doi: 10.1111/j.1539-6924.2011.01703.x.

3. Statistics Canada 2011. Canadian Community Health Survey (CCHS), Cycle 1.1, 2.1, 3.1, 4.1 and 2008, 2009, 2010 Annual Component surveys [Microdata]. Ottawa Ontario: Statistics Canada Health Statistics Division [producer and distributor]: Ottawa Ontario: Data Liberation Initiative [distributor].

4. Statistics Canada 2012. National Population Health Survey (NPHS), Cycle 1-9: Household Component - Longitudinal [Canada] [Microdata]. Ottawa Ontario: Statistics Canada Health Statistics Division [producer and distributor]: Ottawa Ontario: Data Liberation Initiative [distributor].

5. Statistics Canada & Health and Welfare Canada.Canada health survey, 1978-1979 [computer file]. Ottawa, Ont.: Statistics Canada [producer]; Statistics Canada. Data Liberation Initiative [distributor], 1983.

6. Statistics Canada. 1992. General Social Survey,1991 [Canada]: Cycle 6, Health [public use microdata file]. Ottawa, Ontario: Statistics Canada [producer and distributor]. .

7. Statistics Canada. 1986. General Social Survey, Cycle 1, 1985 [Canada]: Health and Social Support [public use microdata file]. Ottawa, Ontario: Statistics Canada [producer and distributor]. .

8. Statistics Canada. Survey of Smoking Habits, 1971-1986 [Canada] [Public-use microdata file(s)]. Ottawa, Ontario: Special Surveys Division, Statistics Canada [producer and distributor]. Ottawa, Ontario: Data Liberation Initiative [distributor].

9. Duffy SW, Field JK, Allgood PC, Seigneurin A. Translation of research results to simple estimates of the likely effect of a lung cancer screening programme in the United Kingdom. 2014;110(7):1834-40. doi: 10.1038/bjc.2014.63.

10. Canadian Human Mortality Database. Department of Demography, Université de Montréal (Canada). Available at [www.demo.umontreal.ca/chmd/](http://www.demo.umontreal.ca/chmd/) (data downloaded on 14-03-2014). [Internet].

11. Levy DT, Romano E, Mumford E. The Relationship of Smoking Cessation to Sociodemographic Characteristics, Smoking Intensity, and Tobacco Control Policies. Nicotine & Tobacco Research. 2005;7(3):387-96. doi: 10.1080/14622200500125443.

12. Fiore MC, Novotny TE, Pierce JP, et al. Methods used to quit smoking in the united states: Do cessation programs help? JAMA. 1990;263(20):2760-5.

13. Hatziandreu EJ, Pierce JP, Fiore MC, Grise V, Novotny TE, Davis RM. The reliability of self-reported cigarette consumption in the United States. American Journal of Public Health. 1989;79(8):1020-3. PubMed PMID: PMC1349899.

14. Warner KE. Possible Increases in the Underreporting of Cigarette Consumption. Journal of the American Statistical Association. 1978;73(362):314-8. doi: 10.2307/2286658.

15. Burns D, Major JM, Shanks TG. Chapter 7: Changes in number of cigarettes smoked per day: cross-sectional and birht cohort analyses using NHIS. 2003. In: Those Who Continue to Smoke Smoking and Tobacco Control Monograph No 15 NIH Pub No 03-5370, September 2003 [Internet].

16. Jeon J, Meza R, Krapcho M, Clarke LD, Byrne J, Levy DT. Chapter 5: Actual and Counterfactual Smoking Prevalence Rates in the U.S. Population via Microsimulation. Risk Analysis. 2012;32:S51-S68. doi: 10.1111/j.1539-6924.2011.01775.x.

17. Rosenberg MA, Feuer EJ, Yu B, Sun J, Henley SJ, Shanks TG, et al. Chapter 3: Cohort Life Tables by Smoking Status, Removing Lung Cancer as a Cause of Death. Risk Analysis. 2012;32:S25-S38. doi: 10.1111/j.1539-6924.2011.01662.x.

18. <https://www.cancercare.on.ca/cms/one.aspx?objectId=121939&contextId=1377>.

19. Thun MJ, Carter BD, Feskanich D, Freedman ND, Prentice R, Lopez AD, et al. 50-Year Trends in Smoking-Related Mortality in the United States. New England Journal of Medicine. 2013;368(4):351-64. doi: doi:10.1056/NEJMsa1211127. PubMed PMID: 23343064.

**Table A: Observed and estimated ever-smoking prevalence at age 30 for male cohorts**

| **Men** | | |
| --- | --- | --- |
| **Cohort** | **Observed ever-smoking prevalence at age 30** | **Estimated ever-smoking prevalence at age 30** |
| 1940-1949 | 61.92% | 61.84% |
| 1950-1959 | 57.78% | 57.75% |
| 1960-1969 | 50.78% | 50.85% |
| **Women** | | |
| **Cohort** | **Observed ever-smoking prevalence at age 30** | **Estimated ever-smoking prevalence at age 30** |
| 1940-1949 | 44.91% | 44.90% |
| 1950-1959 | 46.85% | 46.82% |
| 1960-1969 | 41.01% | 41.03% |

**Table B: Observed and estimated current-, former- and never-smoking prevalences in 2000**

| **Men** | | | | | | | |
| --- | --- | --- | --- | --- | --- | --- | --- |
| **Cohort** | **Observed current-smoking prevalence in 2000** | **Estimated current-smoking prevalence in 2000** | | **Observed former-smoking prevalence in 2000** | **Estimated former-smoking prevalence in 2000** | **Observed never-smoking prevalence in 2000** | **Estimated never-smoking prevalence in 2000** |
| 1940-1949 | 19.13% | 19.62% | | 42.50% | 41.55% | 38.37% | 38.83% |
| 1950-1959 | 24.59% | 25.02% | | 32.56% | 32.61% | 42.85% | 42.37% |
| 1960-1969 | 28.67% | 30.33% | | 20.71% | 20.50% | 50.62% | 49.17% |
| **Women** | | | | | | | |
| **Cohort** | **Observed current-smoking prevalence in 2000** | **Estimated current-smoking prevalence in 2000** | **Observed former-smoking prevalence in 2000** | | **Estimated former-smoking prevalence in 2000** | **Observed never-smoking prevalence in 2000** | **Estimated never-smoking prevalence in 2000** |
| 1940-1949 | 14,62% | 15.18% | 28.89% | | 29.27% | 56.49% | 55.55% |
| 1950-1959 | 20.85% | 20.65% | 24.98% | | 26.10% | 54.17% | 53.26% |
| 1960-1969 | 21.37% | 24.14% | 18.07% | | 16.89% | 60.56% | 58.97% |

**Figure A: Average number of cigarettes per day for ages over 30, by smoking quintile for men born between 1940-1949**

**
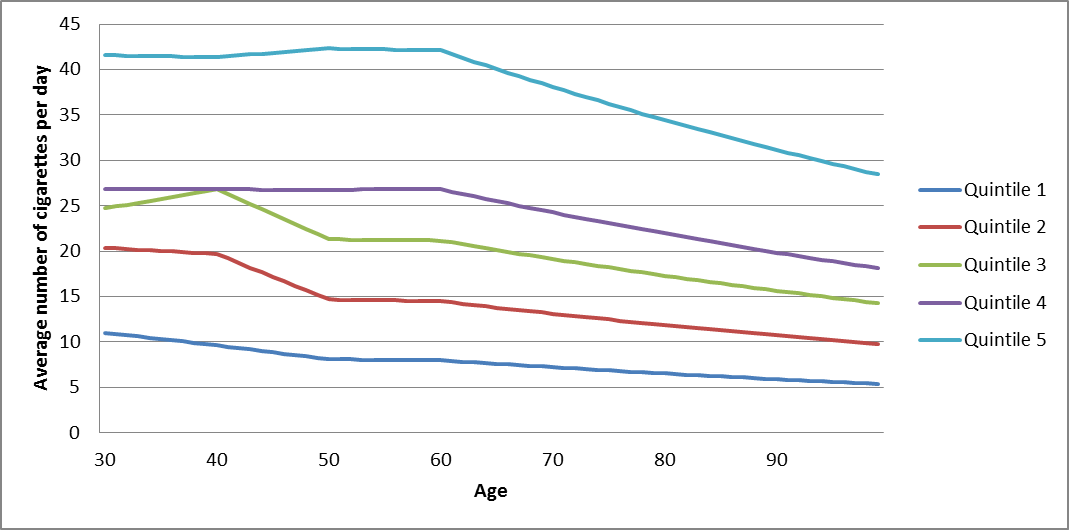
**

**Figure B: Average number of cigarettes per day for ages over 30, by smoking quintile for women born between 1940-1949**

**
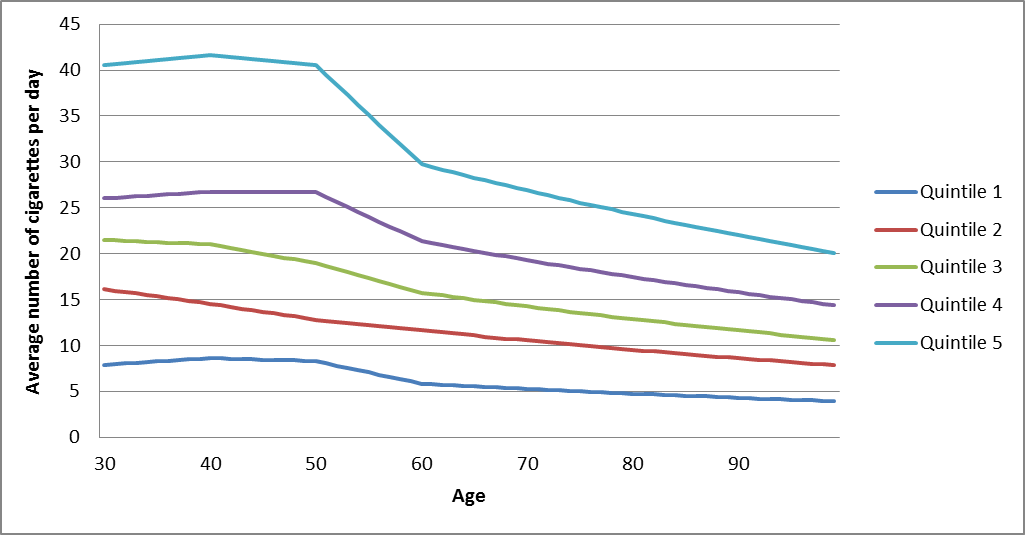
**

**Figure C: Annual probability of dying from all causes for men born in 1955**

**
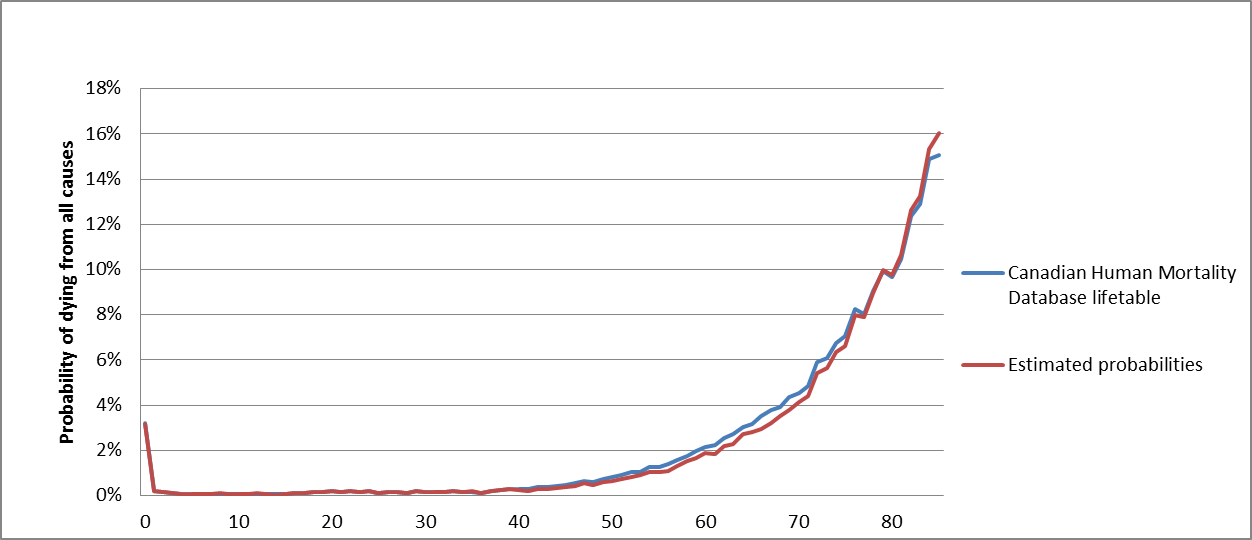
**

**Figure D: Annual probability of dying from all causes for women born in 1955**

**
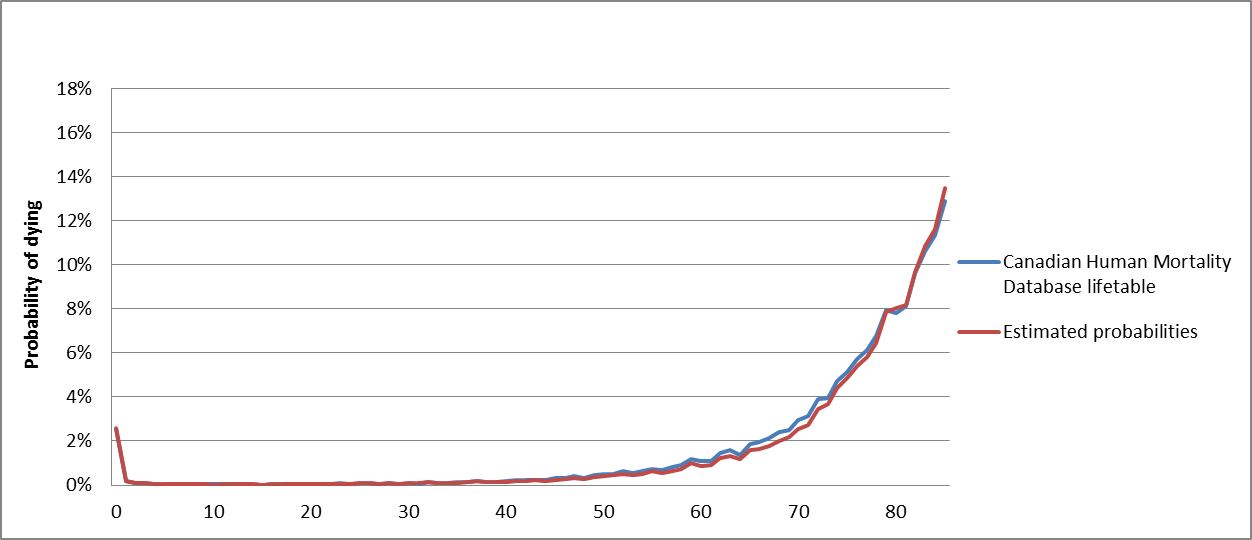
**

**Figure E: Cumulative probability of dying from causes other than lung cancer for never-smokers and current smokers (by smoking quintile) for men born in 1955**

**
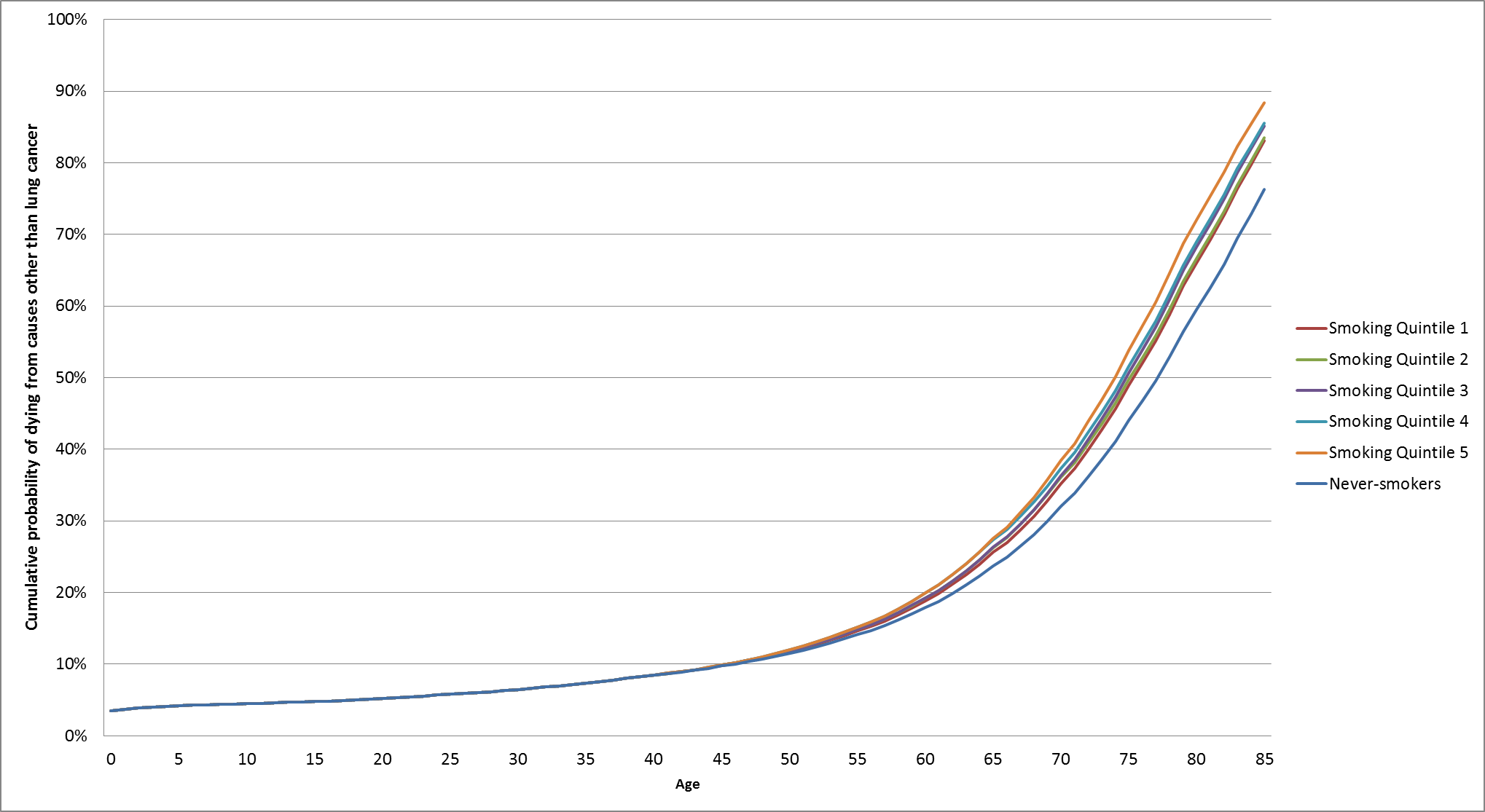
**

**Figure F: Cumulative probability of dying from causes other than lung cancer for never-smokers and current smokers (by smoking quintile) for women born in 1955**

**
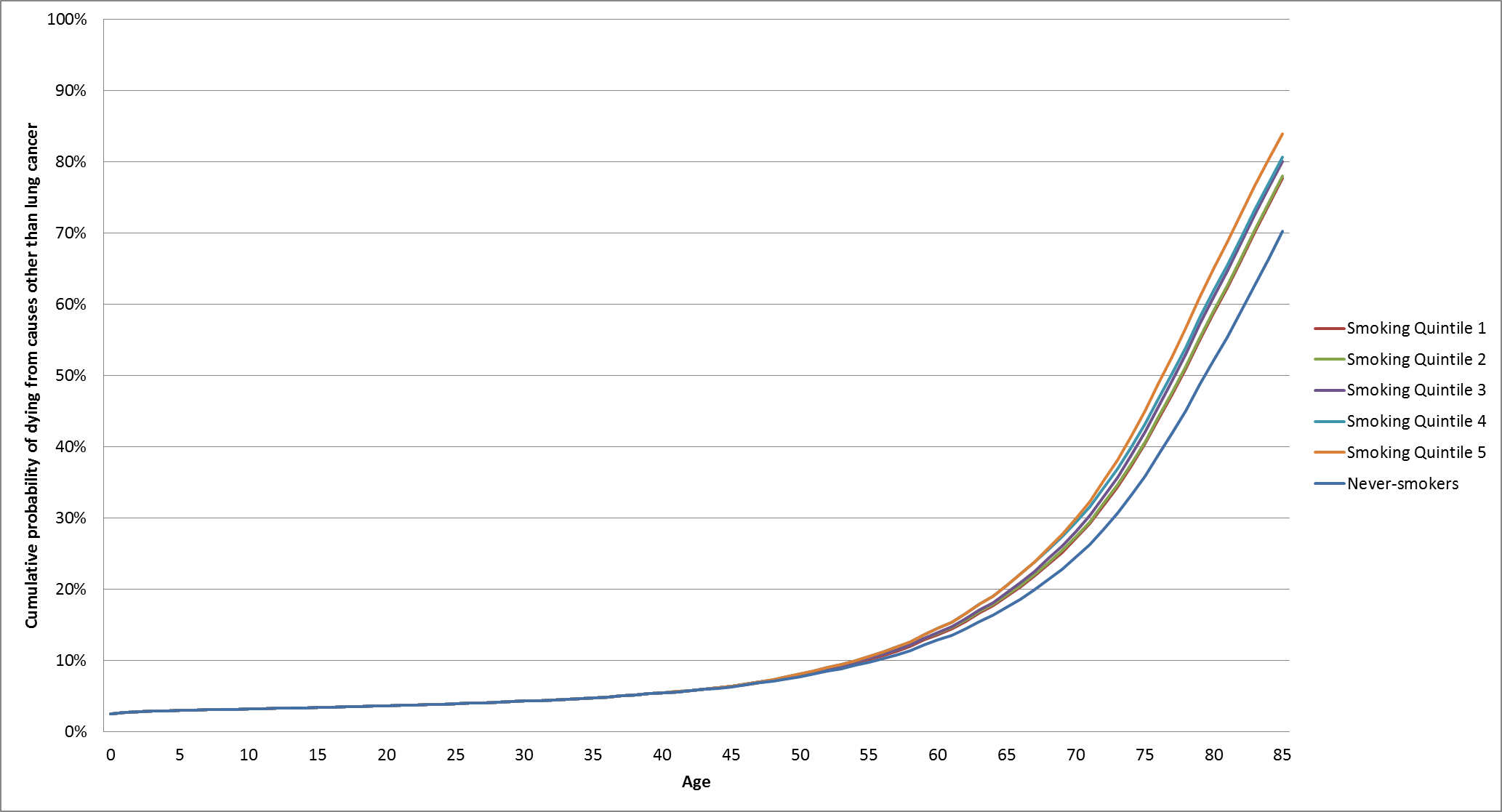
**
