# Supplementary material for: Performance and Cost-Effectiveness of Computed Tomography Lung Cancer Screening Scenarios in a Population-Based Setting: A Microsimulation Modeling Analysis in Ontario, Canada
Source: PLoS Med. 2017 Feb 7;14(2):e1002225. doi: 10.1371/journal.pmed.1002225 (PMC5295664; doi:10.1371/journal.pmed.1002225)

**Supplement S3: Lung cancer incidence in Ontario**

The following sections detail the data and methods used to investigate whether the MISCAN-Lung model can reproduce the observed lung cancer incidence in Ontario, which allows the extrapolation of future lung cancer incidence and investigation of the effects of lung cancer screening.

**Lung cancer incidence**

Data on the incidence of lung cancer (by gender, stage and histology) in the province of Ontario, Canada, was obtained from the Ontario Cancer Registry through an electronic copy located on a secure server at the Institute for Clinical Evaluative Sciences (Toronto, Ontario), for years 2007-2009, for ages 40-74 [1]. Only cancers with a known histology were taken into account, as cancers with an unknown or unspecified histological type could potentially be misdiagnosed metastases from other cancer sites (i.e. 7.74% of cases were excluded).

**Lung cancer stages**

Four tumor stages are distinguished in the Ontario Cancer Registry data, based on the American Joint Committee on Cancer (AJCC) Cancer Staging Manual, 6th edition: stages I, II, III and IV [2]. In the MISCAN-Lung model, six AJCC tumor stages are distinguished, namely: IA, IB, II, IIIA, IIIB and IV. To allow comparisons between the observed lung cancer incidence in the Ontario registry data and the MISCAN-Lung estimates, model outputs for stages IA and IB were combined into stage I and model outputs for stages IIIA and IIIB were combined into stage III.

**Lung cancer histology**

MISCAN-Lung incorporates four histological types, based on the International Classification of Diseases for Oncology Third Edition (ICD-O-3) codes, namely: adenocarcinoma/large cell carcinoma/bronchioloalveolar carcinoma (AD), squamous cell carcinoma (SQ), other non-small cell carcinoma (OTH) and small cell carcinoma (SM) [3,4]. The data from the Ontario Cancer Registry were matched to the definitions used by MISCAN-Lung, using the ICD-O-3 codes.

Five-year survival data by histology, stage and gender were available from the Ontario Cancer Registry and compared to survival data (by stage and histology) obtained from the U.S. Surveillance, Epidemiology, and End Results (SEER) program [5]. The overall survival rates in Ontario and SEER were similar (data not shown). However, due to known differences in survival between stages IA and IB, the survival data from SEER were used, because of availability of more detailed data on survival by stage [6].

**Comparison of MISCAN-Lung estimates to observed data**

Figures A and B compare the proportions of histological types observed in the Ontario Cancer Registry data for ages 40-74, by gender, to the proportions estimated by MISCAN-Lung. Overall, MISCAN-Lung reproduces the observed proportions of histological types for both genders.

Figures C and D compare the proportions of the clinical stages observed for ages 40-74 for lung cancers with a known stage in the Ontario Cancer Registry data, by gender, to the proportions estimated by MISCAN-Lung. Overall, MISCAN-Lung reproduces the observed proportions of clinical stages for each gender, though it somewhat overestimates the proportion of stage I cancers and underestimates the proportion of stage IV cancers for men.

Figures E and F compare the incidence per 100,000 persons by age group and gender, observed in the Ontario registry data to the incidence estimated by MISCAN-Lung. Overall, MISCAN-Lung reproduces the observed incidence well for men, though it somewhat underestimates the incidence at ages 70-74. MISCAN-lung somewhat underestimates the incidence for women across ages 45-69, while it somewhat overestimates the incidence for ages 70-74.

Overall, the MISCAN-Lung model adequately reproduced the overall lung cancer incidence in Ontario for 2007-2009, allowing extrapolation of future lung cancer incidence and investigation of the effects of lung cancer screening.

**References**

1. <https://www.cancercare.on.ca/cms/one.aspx?objectId=121939&contextId=1377>.

2. Greene F.L., Page D.L., Fleming I.D., Fritz A.G., Balch C.M., Haller D.G., et al. eds, for the American Joint Committee on Cancer. AJCC Cancer Staging Manual. 6th ed. New York, NY: Springer-Verlag; 2002.

3. ICD-O-3 SEER Site/Histology Validation List - 12/04/2009 - updated from 2/9/2001 [cited Accessed Jan 12 2012]. Available from: <http://seer.cancer.gov/icd-o-3/>.

4. A. Fritz CP, A. Jack, K. Shanmugaratnam, L. Sobin, D.M. Parkin and S. Whelan. International Classification of Diseases for Oncology third edition. Geneva: World Health Organization; 2000.

5. Surveillance, Epidemiology, and End Results (SEER) Program ([www.seer.cancer.gov](http://www.seer.cancer.gov)) SEER*Stat Database: Incidence - SEER 18 Regs Research Data + Hurricane Katrina Impacted Louisiana Cases, Nov 2012 Sub (1973-2010 varying) - Linked To County Attributes - Total U.S., 1969-2011 Counties, National Cancer Institute, DCCPS, Surveillance Research Program, Surveillance Systems Branch, released April 2013, based on the November 2012 submission.. Accessed March, 29 2014.

6. Groome PA, Bolejack V, Crowley JJ, Kennedy C, Krasnik M, Sobin LH, et al. The IASLC Lung Cancer Staging Project: Validation of the Proposals for Revision of the T, N, and M Descriptors and Consequent Stage Groupings in the Forthcoming (Seventh) Edition of the TNM Classification of Malignant Tumours. Journal of Thoracic Oncology. 2007;2(8):694-705.

**Figure A: Lung cancer histology distributions estimated for men ages 40-74 by the MISCAN-Lung model compared to the observed lung cancer histology distributions in Ontario in 2007-2009**

**
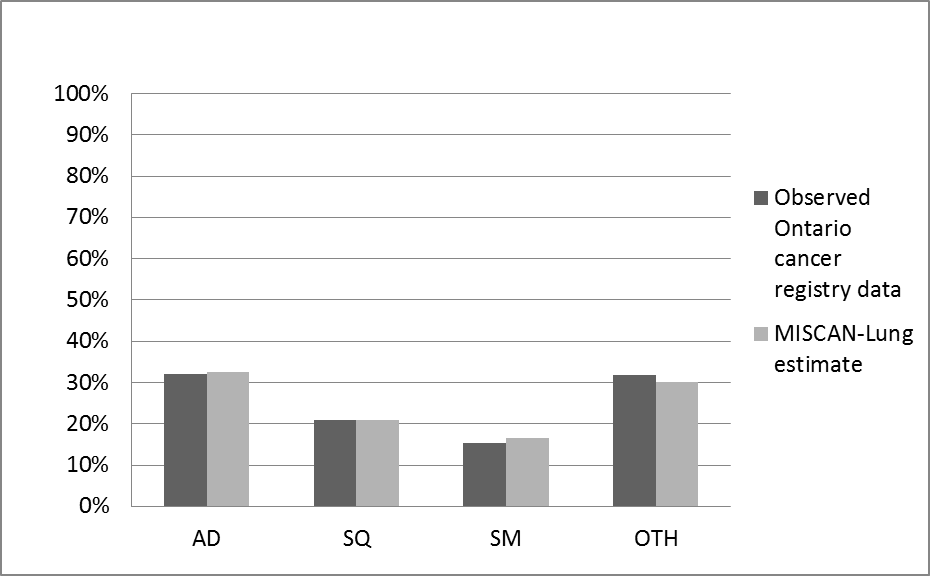
**

**Figure notes:** Abbreviations: adenocarcinoma/large cell carcinoma/ bronchioloalveolar carcinoma (AD), squamous cell carcinoma (SQ), small cell carcinoma (SM), other non–small cell carcinoma (OTH).

**Figure B: Lung cancer histology distributions estimated for women ages 40-74 by the MISCAN-Lung model compared to the observed lung cancer histology distributions in Ontario in 2007-2009**

**
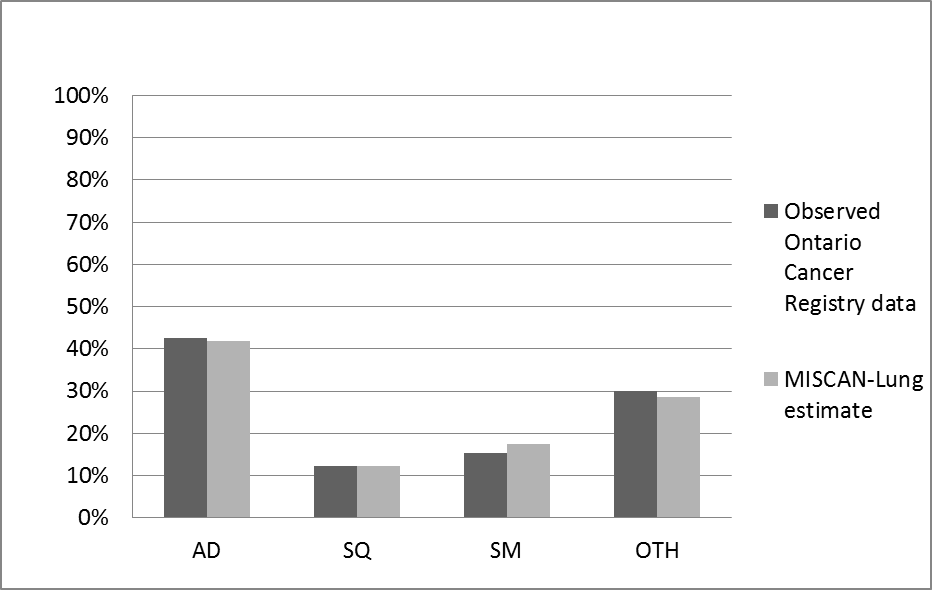
**

**Figure notes:** Abbreviations: adenocarcinoma/large cell carcinoma/ bronchioloalveolar carcinoma (AD), squamous cell carcinoma (SQ), small cell carcinoma (SM), other non–small cell carcinoma (OTH).

**Figure C: Lung cancer stage distributions estimated for men ages 40-74 by the MISCAN-Lung model compared to the observed lung cancer stage distributions in Ontario in 2007-2009**

**
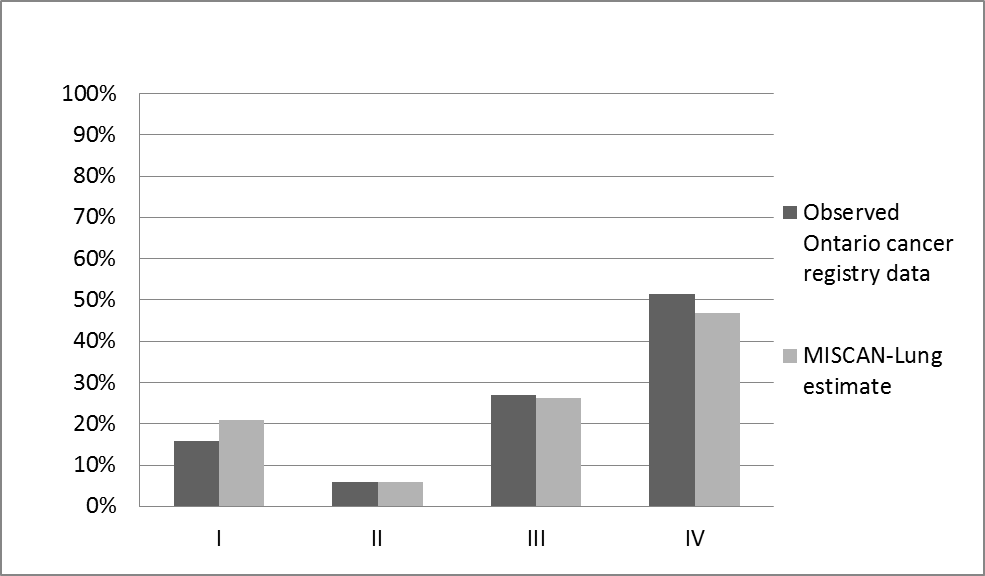
**

**Figure D: Lung cancer stage distributions estimated for women ages 40-74 by the MISCAN-Lung model compared to the observed lung cancer stage distributions in Ontario in 2007-2009**


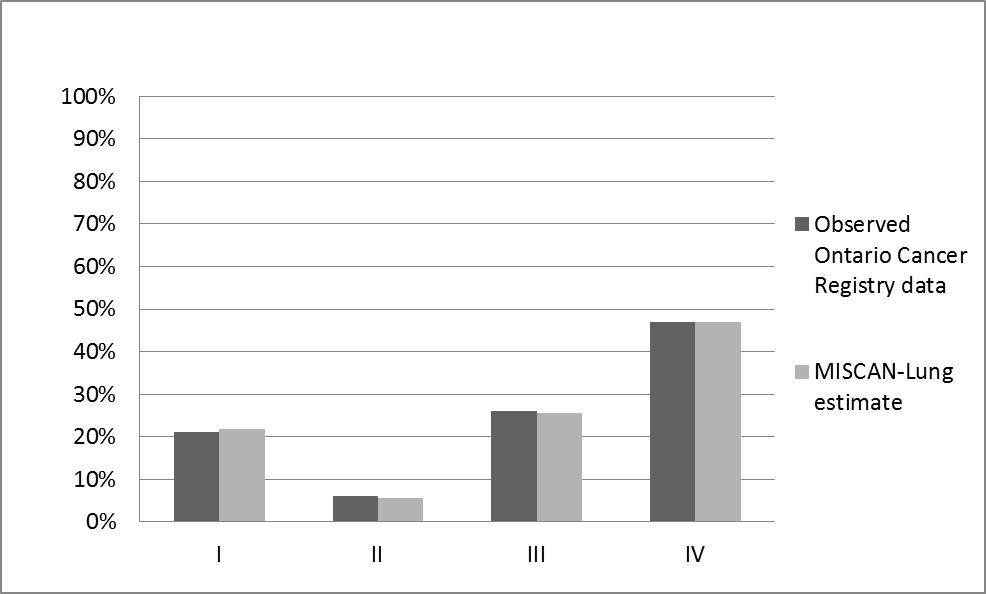


**Figure E: Lung cancer incidence per 100,000 estimated for men ages 40-74 by the MISCAN-Lung model compared to the observed lung cancer incidence in Ontario in 2007-2009**

**
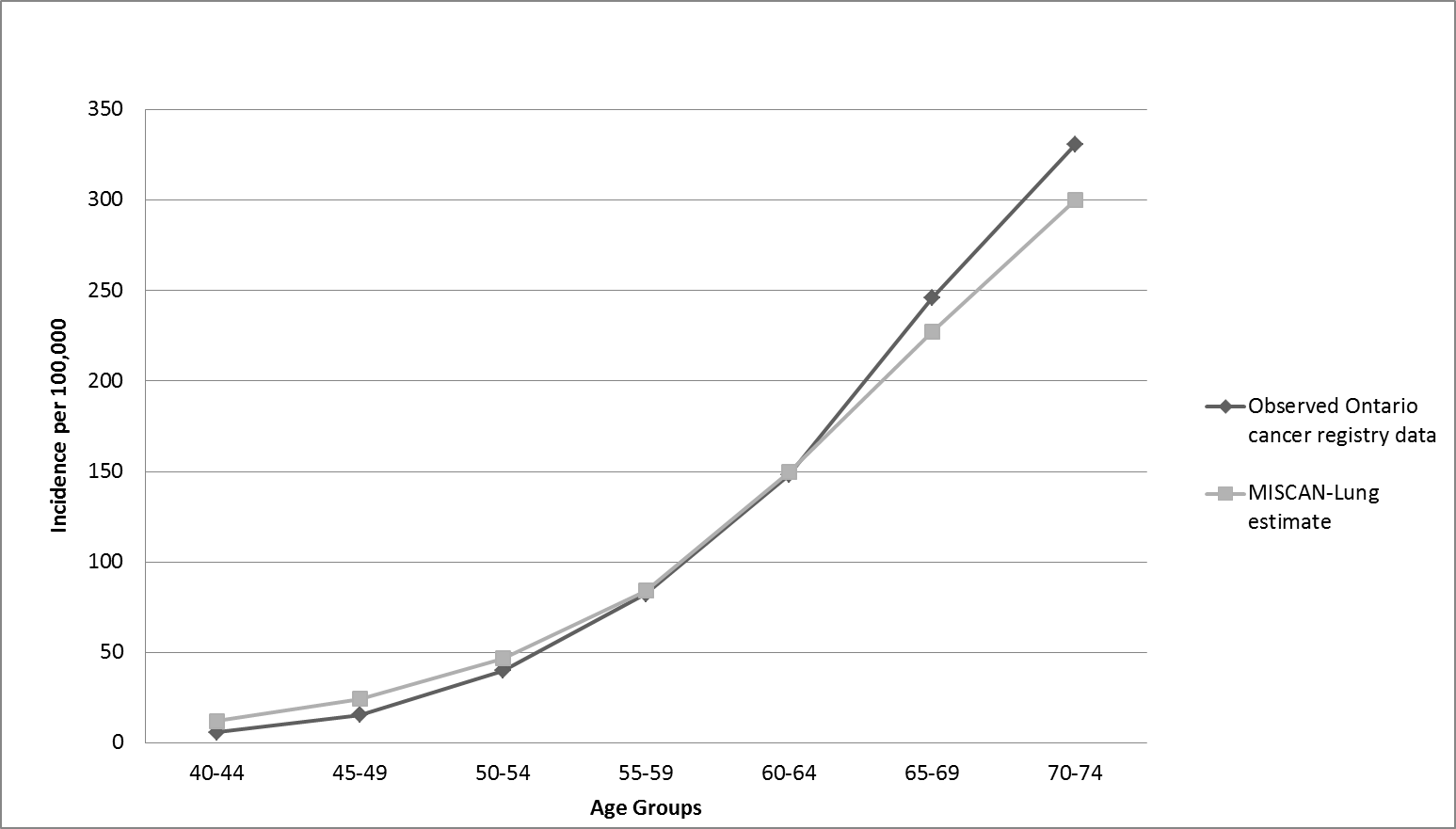
**

**Figure F: Lung cancer incidence per 100,000 estimated for women ages 40-74 by the MISCAN-Lung model compared to the observed lung cancer incidence in Ontario in 2007-2009**


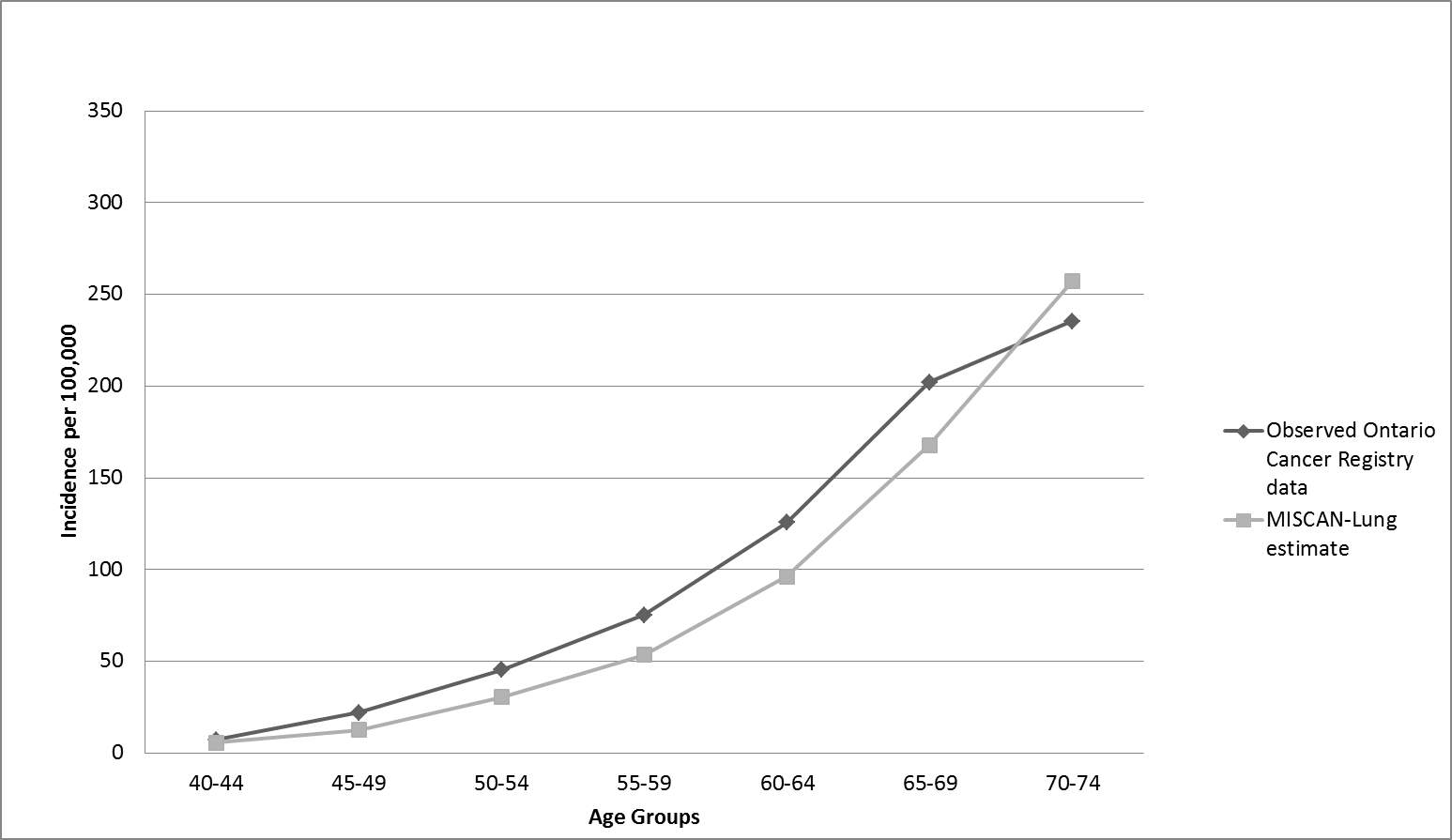

Supplement: S3 Text — (DOCX) [file pmed.1002225.s004.docx]
