# Supplementary material for: Performance and Cost-Effectiveness of Computed Tomography Lung Cancer Screening Scenarios in a Population-Based Setting: A Microsimulation Modeling Analysis in Ontario, Canada
Source: PLoS Med. 2017 Feb 7;14(2):e1002225. doi: 10.1371/journal.pmed.1002225 (PMC5295664; doi:10.1371/journal.pmed.1002225)
Supplement: S4 Text — (DOCX) [file pmed.1002225.s005.docx]

**Supplement S4: Screening related follow-up procedures**

The MISCAN-Lung model incorporates the following screening related follow-up procedures: false-positive results, follow-up CT examinations, biopsies and surgeries not related to lung cancer as a result of screening. The following sections detail the methods and assumptions used to incorporate these screening related follow-up procedures in the MISCAN-Lung model.

**False-positive results**

Each individual in the model who attends a round of screening, in whom lung cancer is not detected by screening in that round, has a chance to receive a false-positive test result. The probability of receiving a false-positive test result was based on individual-level data from the computed tomography (CT) arm of the National Lung Screening Trial (NLST). For each screening round in the CT arm of the NLST, the number of positive screening test results and the number of persons with screen-detected lung cancer were determined. The number of false-positive test results in each round of the CT arm of the NLST was defined as the number of positive screening test results minus the number of persons with screen-detected lung cancer in that round. The probability to receive a false-positive screening test result in each round of the CT arm of the NLST was then defined as follows:

$$P\left( False positive test result in round X \right|Lung cancer not detected by screening in round X)=\frac{(Number of positive test results in round X-Number of persons with screen detected lung cancer in round X)}{(Number of screens in round X-Number of persons with screen detected lung cancer in round X)}$$

The probability that an individual in the model who attends screening, in whom lung cancer is not detected by screening in that round, receives a false-positive test result is assumed to be equal to the probability observed in the corresponding round of the CT arm of the NLST. For every screening round attended after the third, this probability is assumed to be equal to that of the third screening round of the NLST. The corresponding probabilities for each round are noted in Table A.

**Follow-up CT-examinations**

Each individual in the model in whom lung cancer is detected (through screening or clinical presentation of symptoms) receives a CT examination (in addition to a biopsy, as detailed in the section on biopsies) to confirm the diagnosis of cancer, which is incorporated into the attributable costs. In addition, each individual who attends a screening round, in whom lung cancer is not detected by screening in that round, has a chance to receive a number of follow-up CT examinations as a result of screening. The probability in the model to receive at least one follow-up CT examination and the average number of follow-up CT examinations received by persons without screen-detected lung cancer were based on individual-level data from the CT arm of the NLST. Table B lists the follow-up procedures in the NLST dataset classified as follow-up CT examinations for this investigation. For each screening round in the CT arm of the NLST, the number of persons without a screen-detected lung cancer in that round who received at least one screen-related follow-up CT examination was determined. The probability that an individual without screen-detected lung cancer in that round receives at least one screen-related follow-up CT examination was then defined as follows for each round of the CT arm of the NLST:

$$P\left( At least one followup CT examination in round X \right|Lung cancer not detected by screening in round X)=\frac{(Number of persons with at least one followup CT examination without screen detected lung cancer in round X)}{(Number of screens in round X-Number of persons with screen detected lung cancer in round X)}$$

The average number of screen-related follow-up CT examinations per person, in whom lung cancer was not detected by screening, who received at least one follow-up CT examination was then defined as follows for each round of the CT arm of the NLST:

$$\left( \begin{aligned} Average number of followup CT examinations \\ per person who received at least one \\ CT followup examination \\ in round X \end{aligned} \right|Lung cancer not detected by screening in round X)$$

$$=\frac{(Number of followup CT examinations for persons without screen detected lung cancer in round X)}{(Number of persons with at least one followup CT examination without screen detected lung cancer in round X)}$$

The probability that an individual in the model who attends screening, in whom lung cancer is not detected by screening in that round, receives at least one follow-up CT examination is assumed to be equal to the probability observed in the corresponding round of the CT arm of the NLST. The average number of follow-up CT examinations per person, in whom lung cancer is not detected by screening, who receives at least one CT examination is assumed to be equal to the average number of CT examinations per person examined in the corresponding round of the CT arm of the NLST. For every screening round attended after the third, the probability to receive a follow-up CT examination and the average number of follow-up CT examinations were assumed to be equal to those of the third screening round of NLST. The corresponding probabilities and average number of follow-up CT examinations per person examined are described in Table A.

**Biopsies/bronchoscopies**

Every person in the model in whom lung cancer is detected (through screening or clinical presentation of symptoms) receives a biopsy/bronchoscopy (in addition to a CT examination, as detailed in the section on CT examinations) to confirm the diagnosis of cancer, which is incorporated into the attributable costs. However, individuals who attend screening (in whom lung cancer is not detected by screening) also have a chance to receive a biopsy/bronchoscopy as a result of screening. The probability of receiving a biopsy/bronchoscopy was based on individual-level data from the CT arm of the NLST. Table C lists the follow-up procedures in the NLST dataset classified as biopsies for this investigation. For each screening round in the CT arm of the NLST, the number of persons, in whom lung cancer was not detected by screening, that received at least one biopsy related to the screening round was determined. Biopsies/bronchoscopies performed on days on which a surgical procedure was performed and cytology procedures performed on the same day as another biopsy/bronchoscopy procedure were not counted as separate events.

The probability for a person, in whom lung cancer was not detected by screening, to receive a screen-related biopsy/bronchoscopy was then defined as follows for each round of the CT arm of the NLST:

$$P\left( Screenrelated biopsy in round X \right|Lung cancer not detected by screening in round X)=\frac{(Number of persons with at least one screenrelated biopsy/bronchoscopy without screen detected lung cancer in round X)}{(Number of screens in round X-Number of persons with screen detected lung cancer in round X)}$$

The probability that an individual in the model who attends screening, in whom lung cancer is not detected by screening in that round, receives a biopsy/bronchoscopy related to screening is assumed to be equal to the probability observed in the corresponding round of the CT arm of the NLST. For every screening round attended after the third, this probability is assumed to be equal to that of the third screening round of NLST. The corresponding probabilities for each round are noted in Table A.

**Surgeries not related to lung cancer as a result of screening**

Individuals in the model who attend screening (in whom lung cancer is not detected by screening) have a chance to receive a surgery not related to lung cancer as a result of the screening**.** The probability of receiving a surgery not related to lung cancer as a result of screening was based on individual-level data from the CT arm of the NLST. Table D lists the follow-up procedures in the NLST dataset classified as surgical procedures for this investigation. For each screening round in the CT arm of the NLST, the number of persons who received at least one surgical procedure not related to a diagnosis of lung cancer as a result of screening was determined.

The probability for a person, in whom lung cancer was not detected by screening, to receive a screen-related surgery not related to lung cancer was then defined as follows for each round of the CT arm of the NLST:

$$P\left( Screen related surgery not related to lung cancer in round X \right|Lung cancer not detected by screening in round X)=\frac{(Number of persons with at least one screen related surgery without screen detected lung cancer in round X)}{(Number of screens in round X-Number of persons with screen detected lung cancer in round X)}$$

The probability that an individual in the model who attends screening, in whom lung cancer is not detected by screening in that round, receives a surgical procedure not related to lung cancer is assumed to be equal to the probability observed in the corresponding round of the CT arm of the NLST. For every screening round attended after the third, this probability assumed to be equal to that of the third screening round of NLST. The corresponding probabilities for each round are noted in Table A.

**Table A**: **Probabilities of adverse outcomes due to screening in the MISCAN-Lung model given that lung cancer is not detected by screening**

| **Screening round** | **Probability to receive a false-positive result** | **Probability to receive a follow-up CT examination** | **Average number of follow-up CT examinations received per person receiving follow-up CT examinations** | **Probability to receive a biopsy/bronchoscopy** | **Probability to receive a surgery not related to lung as a result of screening** |
| --- | --- | --- | --- | --- | --- |
| First attended screening | 26.58% | 19.27% | 1.45 | 0.76% | 0.35% |
| Second attended screening | 27.44% | 7.83% | 1.32 | 0.39% | 0.20% |
| Third attended screening and subsequent screens | 16.05% | 6.37% | 1.69 | 0.39% | 0.18% |

**Table B**: **Procedures in the NLST dataset classified as follow-up CT scans**

| **Procedures** |
| --- |
| "CT- Abdomen and pelvis"  "CT - Chest, limited thin section of nodule"  "CT - Diagnostic chest"  "CT - Chest and abdomen"  "CT - Brain"  "CT - Abdomen (or liver)"  "CT - Chest, low dose spiral"  "CT - Chest, abdomen, and pelvis"  "CT - Other (specify)"  "CT - Chest, plus nodule densitometry"  "CT- Chest limited thin section of entire lung"  "Radionuclide scan - Fusion PET/CT scan" |

**Table C: Procedures classified as biopsies/bronchoscopies in the NLST dataset**

| **Procedures** |
| --- |
| “Biopsy - Endobronchial"  "Biopsy – Other"  "Biopsy - Percutaneous adrenal"  "Cytology - Bronchoscopic"  "Biopsy - Percutaneous Liver"  "Biopsy - Open Surgical"  "Biopsy - Percutaneous transthoracic yielding histology"  "Cytology -Percutaneous transthoracic"  "Biopsy - Lymph node - other"  "Biopsy - Transbronchial"  "Bronchoscopy without biopsy or cytology"  "Cytology – Other”  "Biopsy - Lymph node - scalene nodes"  "Biopsy - Thoracoscopic"  "Cytology - Sputum" |

**Table D**: **Procedures classified as non-lung cancer related surgical procedures (if not linked to the diagnosis of lung cancer)**

| **Procedures** |
| --- |
| "Mediastinoscopy/Mediastinotomy"  "Thoracotomy"  "Lymphadenectomy/lymph node sampling"  "Resection"  “Thoracentesis"  “Thoracoscopy without Biopsy"  "Thoracoscopy" |
