# Supplementary material for: Performance and Cost-Effectiveness of Computed Tomography Lung Cancer Screening Scenarios in a Population-Based Setting: A Microsimulation Modeling Analysis in Ontario, Canada
Source: PLoS Med. 2017 Feb 7;14(2):e1002225. doi: 10.1371/journal.pmed.1002225 (PMC5295664; doi:10.1371/journal.pmed.1002225)
Supplement: S5 Text — (DOCX) [file pmed.1002225.s006.docx]

**Supplement S5: Impact of sensitivity analyses**

The following section details the characteristics, costs, life-years gained and cost-effectiveness of the scenarios on the efficient frontier of each sensitivity analysis. Tables A-C show the effects of varying the attendance rates. Tables D-E show the effects of halving or doubling the attributable costs. Finally, Tables F-G show the effects of halving or doubling the CT examination costs.

Table H shows the presence of the scenarios that were on the efficient frontier in the base-case analysis across the efficient frontiers of the sensitivity analyses. Table H shows that the cost-effectiveness estimates for scenarios #2, #3 and # 9-11 in the base-case were the most insensitive to changes in assumptions, as they were part of the efficient frontier across 85.71-100% of the sensitivity analyses.

| **Scenario # on current efficient frontier** | **Starting age of screening** | **Stopping age of screening** | **Screening interval** | **Maximum number of years since cessation** | **Cumulative smoking criteria** | **Exclusion from further screening invitations after reaching the maximum number of years since cessation** | **Discounted costs per 100,000** | **Discounted life-years gained per 100,000** | **Costs per life-year gained (discounted) / Average cost-effectiveness ratio (ACER) compared to no screening** | **Incremental cost-effectiveness ratio (ICER) compared to the previous efficient scenario on the current frontier** | **Scenario present on efficient frontier in the base case analyses?** |
| --- | --- | --- | --- | --- | --- | --- | --- | --- | --- | --- | --- |
| #1 | 55 | 75 | Annual | 10 | 40 pack-years (NLST-like) | Yes | $42,753,778 | 1,049 | $40,757 | - | Yes  (Scenario #2) |
| #2 | 55 | 75 | Annual | 10 | 30 pack-years (NLST-like) | Yes | $52,196,709 | 1,224 | $42,630 | $53,830 | Yes  (Scenario #3) |
| #3 | 55 | 75 | Annual | 20 | 30 years of smoking at least 15 cigarettes per day **or** 35 years of smoking at least 10 cigarettes per day (NELSON-like) | No | $75,079,668 | 1,596 | $47,035 | $61,544 | No |
| #4 | 50 | 75 | Annual | 20 | 30 years of smoking at least 15 cigarettes per day **or** 35 years of smoking at least 10 cigarettes per day (NELSON-like) | No | $79,653,298 | 1,667 | $47,779 | $64,518 | No |
| #5 | 50 | 80 | Annual | 20 | 30 years of smoking at least 15 cigarettes per day **or** 35 years of smoking at least 10 cigarettes per day (NELSON-like) | No | $91,761,957 | 1,845 | $49,739 | $68,122 | Yes  (Scenario #9) |
| #6 | 50 | 80 | Annual | 20 | 25 years of smoking at least 15 cigarettes per day **or** 30 years of smoking at least 10 cigarettes per day (NELSON-like) | No | $111,914,998 | 2,059 | $54,352 | $94,081 | Yes  (Scenario #10) |
| #7 | 50 | 80 | Annual | 20 | 25 years of smoking at least 10 cigarettes per day **or** 30 years of smoking at least 5 cigarettes per day (NELSON-like) | No | $140,231,037 | 2,297 | $61,039 | $118,824 | Yes  (Scenario # 11) |

**Table A: Cost-effectiveness estimates for lung cancer screening scenarios on the efficient frontier when assuming high attendance rates (64% overall compliance).**

**Table B: Cost-effectiveness estimates for lung cancer screening scenarios on the efficient frontier when assuming average attendance rates (55% overall compliance).**

| **Scenario # on current efficient frontier** | **Starting age of screening** | **Stopping age of screening** | **Screening interval** | **Maximum number of years since cessation** | **Cumulative smoking criteria** | **Exclusion from further screening invitations after reaching the maximum number of years since cessation** | **Discounted costs per 100,000** | **Discounted life-years gained per 100,000** | **Costs per life-year gained (discounted) / Average cost-effectiveness ratio (ACER) compared to no screening** | **Incremental cost-effectiveness ratio (ICER) compared to the previous efficient scenario on the current frontier** | **Scenario present on efficient frontier in the base case analyses?** |
| --- | --- | --- | --- | --- | --- | --- | --- | --- | --- | --- | --- |
| #1 | 55 | 75 | Annual | 10 | 40 pack-years (NLST-like) | Yes | $38,598,562 | 911 | $42,373 | - | Yes (Scenario # 2) |
| #2 | 55 | 75 | Annual | 10 | 30 pack-years (NLST-like) | Yes | $46,793,945 | 1,064 | $43,971 | $53,470 | Yes (Scenario #3) |
| #3 | 55 | 75 | Annual | 20 | 30 years of smoking at least 15 cigarettes per day **or** 35 years of smoking at least 10 cigarettes per day (NELSON-like) | Yes | $64,034,640 | 1,345 | $47,594 | $61,304 | No |
| #4 | 55 | 75 | Annual | 20 | 30 years of smoking at least 15 cigarettes per day **or** 35 years of smoking at least 10 cigarettes per day (NELSON-like) | No | $66,780,467 | 1,390 | $48,049 | $61,835 | No |
| #5 | 50 | 75 | Annual | 20 | 30 years of smoking at least 15 cigarettes per day **or** 35 years of smoking at least 10 cigarettes per day (NELSON-like) | No | $70,788,400 | 1,450 | $48,824 | $66,768 | No |
| #6 | 50 | 80 | Annual | 20 | 30 years of smoking at least 15 cigarettes per day **or** 35 years of smoking at least 10 cigarettes per day (NELSON-like) | No | $81,603,480 | 1,606 | $50,797 | $69,058 | Yes (Scenario #9) |
| #7 | 50 | 80 | Annual | 20 | 25 years of smoking at least 15 cigarettes per day **or** 30 years of smoking at least 10 cigarettes per day (NELSON-like) | No | $99,166,673 | 1,791 | $55,369 | $95,172 | Yes (Scenario #10) |
| #8 | 50 | 80 | Annual | 20 | 25 years of smoking at least 10 cigarettes per day **or** 30 years of smoking at least 5 cigarettes per day (NELSON-like) | No | $123,825,644 | 2,003 | $61,828 | $116,460 | Yes (Scenario #11) |

**Table C: Cost-effectiveness estimates for lung cancer screening scenarios on the efficient frontier when assuming low attendance rates (33% overall compliance).**

| **Scenario # on current efficient frontier** | **Starting age of screening** | **Stopping age of screening** | **Screening interval** | **Maximum number of years since cessation** | **Cumulative smoking criteria** | **Exclusion from further screening invitations after reaching the maximum number of years since cessation** | **Discounted costs per 100,000** | **Discounted life-years gained per 100,000** | **Costs per life-year gained (discounted) / Average cost-effectiveness ratio (ACER) compared to no screening** | **Incremental cost-effectiveness ratio (ICER) compared to the previous efficient scenario on the current frontier** | **Scenario present on efficient frontier in the base case analyses?** |
| --- | --- | --- | --- | --- | --- | --- | --- | --- | --- | --- | --- |
| #1 | 55 | 75 | Annual | 10 | 30 pack-years (NLST-like) | Yes | $31,451,475 | 605 | $52,023 | - | Yes (Scenario #3) |
| #2 | 55 | 75 | Annual | 10 | 30 pack-years (NLST-like) | No | $38,205,270 | 717 | $53,306 | $ 60,224 | No |
| #3 | 55 | 75 | Annual | 15 | 30 years of smoking at least 15 cigarettes per day **or** 35 years of smoking at least 10 cigarettes per day (NELSON-like) | No | $42,416,487 | 785 | $54,043 | $ 61,793 | No |
| #4 | 55 | 75 | Annual | 20 | 30 years of smoking at least 15 cigarettes per day **or** 35 years of smoking at least 10 cigarettes per day (NELSON-like) | No | $43,306,130 | 799 | $54,185 | $ 61,961 | No |
| #5 | 50 | 75 | Annual | 20 | 30 years of smoking at least 15 cigarettes per day **or** 35 years of smoking at least 10 cigarettes per day (NELSON-like) | No | $45,749,850 | 838 | $54,620 | $ 63,688 | No |
| #6 | 50 | 80 | Annual | 20 | 30 years of smoking at least 15 cigarettes per day **or** 35 years of smoking at least 10 cigarettes per day (NELSON-like) | No | $52,709,093 | 935 | $56,386 | $ 71,596 | Yes (Scenario #9) |
| #7 | 50 | 80 | Annual | 20 | 25 years of smoking at least 15 cigarettes per day **or** 30 years of smoking at least 10 cigarettes per day (NELSON-like) | No | $63,053,644 | 1,043 | $60,483 | $ 96,041 | Yes (Scenario #10) |
| #8 | 50 | 80 | Annual | 20 | 25 years of smoking at least 10 cigarettes per day **or** 30 years of smoking at least 5 cigarettes per day (NELSON-like) | No | $77,514,316 | 1,166 | $66,455 | $ 116,703 | Yes (Scenario #11) |

**Table D: Cost-effectiveness estimates for lung cancer screening scenarios on the efficient frontier when assuming 50% lower attributable costs.**

| **Scenario # on current efficient frontier** | **Starting age of screening** | **Stopping age of screening** | **Screening interval** | **Maximum number of years since cessation** | **Cumulative smoking criteria** | **Exclusion from further screening invitations after reaching the maximum number of years since cessation** | **Discounted costs per 100,000** | **Discounted life-years gained per 100,000** | **Costs per life-year gained (discounted) / Average cost-effectiveness ratio (ACER) compared to no screening** | **Incremental cost-effectiveness ratio (ICER) compared to the previous efficient scenario on the current frontier** | **Scenario present on efficient frontier in the base case analyses?** |
| --- | --- | --- | --- | --- | --- | --- | --- | --- | --- | --- | --- |
| #1 | 55 | 75 | Biennial | 10 | 40 pack-years (NLST-like) | Yes | $33,992,471 | 1,002 | $33,910 | - | No |
| #2 | 60 | 75 | Annual | 10 | 40 pack-years (NLST-like) | Yes | $43,525,926 | 1,276 | $34,113 | $34,858 | Yes (Scenario #1) |
| #3 | 55 | 75 | Annual | 10 | 40 pack-years (NLST-like) | Yes | $51,688,904 | 1,489 | $34,705 | $38,240 | Yes (Scenario #2) |
| #4 | 55 | 75 | Annual | 10 | 30 pack-years (NLST-like) | Yes | $65,098,514 | 1,746 | $37,288 | $52,289 | Yes (Scenario #3) |
| #5 | 55 | 80 | Annual | 10 | 30 pack-years (NLST-like) | Yes | $69,954,321 | 1,834 | $38,146 | $55,166 | Yes (Scenario #4) |
| #6 | 55 | 80 | Annual | 15 | 30 pack-years (NLST-like) | Yes | $85,678,445 | 2,088 | $41,039 | $61,940 | Yes (Scenario #6) |
| #7 | 55 | 80 | Annual | 20 | 30 years of smoking at least 15 cigarettes per day **or** 35 years of smoking at least 10 cigarettes per day (NELSON-like) | Yes | $102,913,644 | 2,359 | $43,633 | $63,627 | Yes (Scenario #7) |
| #8 | 55 | 80 | Annual | 20 | 30 years of smoking at least 15 cigarettes per day **or** 35 years of smoking at least 10 cigarettes per day (NELSON-like) | No | $112,455,785 | 2,500 | $44,989 | $67,654 | Yes (Scenario #8) |
| #9 | 50 | 80 | Annual | 20 | 30 years of smoking at least 15 cigarettes per day **or** 35 years of smoking at least 10 cigarettes per day (NELSON-like) | No | $119,148,287 | 2,592 | $45,970 | $72,560 | Yes (Scenario #9) |
| #10 | 50 | 80 | Annual | 20 | 25 years of smoking at least 15 cigarettes per day **or** 30 years of smoking at least 10 cigarettes per day (NELSON-like) | No | $149,041,280 | 2,877 | $51,810 | $104,968 | Yes (Scenario #10) |
| #11 | 50 | 80 | Annual | 20 | 25 years of smoking at least 10 cigarettes per day **or** 30 years of smoking at least 5 cigarettes per day (NELSON-like) | No | $191,165,730 | 3,214 | $59,471 | $124,705 | Yes (Scenario #11) |

**Table E: Cost-effectiveness estimates for lung cancer screening scenarios on the efficient frontier when assuming 50% higher attributable costs.**

| **Scenario # on current efficient frontier** | **Starting age of screening** | **Stopping age of screening** | **Screening interval** | **Maximum number of years since cessation** | **Cumulative smoking criteria** | **Exclusion from further screening invitations after reaching the maximum number of years since cessation** | **Discounted costs per 100,000** | **Discounted life-years gained per 100,000** | **Costs per life-year gained (discounted) / Average cost-effectiveness ratio (ACER) compared to no screening** | **Incremental cost-effectiveness ratio (ICER) compared to the previous efficient scenario on the current frontier** | **Scenario present on efficient frontier in the base case analyses?** |
| --- | --- | --- | --- | --- | --- | --- | --- | --- | --- | --- | --- |
| #1 | 55 | 75 | Annual | 10 | 40 pack-years (NLST-like) | Yes | $72,272,006 | 1,489 | $48,525 | - | Yes (Scenario #2) |
| #2 | 55 | 75 | Annual | 10 | 30 pack-years (NLST-like) | Yes | $88,738,235 | 1,746 | $50,828 | $64,208 | Yes (Scenario #3) |
| #3 | 55 | 75 | Annual | 15 | 30 years of smoking at least 15 cigarettes per day **or** 35 years of smoking at least 10 cigarettes per day (NELSON-like) | Yes | $111,723,824 | 2,050 | $54,487 | $75,455 | No |
| #4 | 55 | 75 | Annual | 20 | 30 years of smoking at least 15 cigarettes per day **or** 35 years of smoking at least 10 cigarettes per day (NELSON-like) | Yes | $122,665,084 | 2,192 | $55,952 | $77,138 | No |
| #5 | 55 | 75 | Annual | 20 | 30 years of smoking at least 15 cigarettes per day **or** 35 years of smoking at least 10 cigarettes per day (NELSON-like) | No | $128,075,204 | 2,261 | $56,657 | $79,299 | No |
| #6 | 50 | 75 | Annual | 20 | 30 years of smoking at least 15 cigarettes per day **or** 35 years of smoking at least 10 cigarettes per day (NELSON-like) | No | $135,555,605 | 2,354 | $57,594 | $80,335 | No |
| #7 | 50 | 80 | Annual | 20 | 30 years of smoking at least 15 cigarettes per day **or** 35 years of smoking at least 10 cigarettes per day (NELSON-like) | No | $157,493,361 | 2,592 | $60,764 | $92,085 | Yes (Scenario #9) |
| #8 | 50 | 80 | Annual | 20 | 25 years of smoking at least 15 cigarettes per day **or** 30 years of smoking at least 10 cigarettes per day (NELSON-like) | No | $190,901,753 | 2,877 | $66,362 | $117,312 | Yes (Scenario #10) |
| #9 | 50 | 80 | Annual | 20 | 25 years of smoking at least 10 cigarettes per day **or** 30 years of smoking at least 5 cigarettes per day (NELSON-like) | No | $237,777,957 | 3,214 | $73,971 | $138,772 | Yes (Scenario #11) |

**Table F: Cost-effectiveness estimates for lung cancer screening scenarios on the efficient frontier when assuming 50% lower CT examination costs.**

| **Scenario # on current efficient frontier** | **Starting age of screening** | **Stopping age of screening** | **Screening interval** | **Maximum number of years since cessation** | **Cumulative smoking criteria** | **Exclusion from further screening invitations after reaching the maximum number of years since cessation** | **Discounted costs per 100,000** | **Discounted life-years gained per 100,000** | **Costs per life-year gained (discounted) / Average cost-effectiveness ratio (ACER) compared to no screening** | **Incremental cost-effectiveness ratio (ICER) compared to the previous efficient scenario on the current frontier** | **Scenario present on efficient frontier in the base case analyses?** |
| --- | --- | --- | --- | --- | --- | --- | --- | --- | --- | --- | --- |
| #1 | 55 | 75 | Annual | 10 | 40 pack-years (NLST-like) | Yes | $42,641,746 | 1,489 | $28,630 | - | Yes (Scenario #2) |
| #2 | 55 | 75 | Annual | 10 | 30 pack-years (NLST-like) | Yes | $51,297,788 | 1,746 | $29,383 | $33,753 | Yes (Scenario #3) |
| #3 | 55 | 75 | Annual | 15 | 30 years of smoking at least 15 cigarettes per day **or** 35 years of smoking at least 10 cigarettes per day (NELSON-like) | Yes | $63,374,225 | 2,050 | $30,907 | $39,644 | No |
| #4 | 55 | 75 | Annual | 20 | 30 years of smoking at least 15 cigarettes per day **or** 35 years of smoking at least 10 cigarettes per day (NELSON-like) | Yes | $69,111,248 | 2,192 | $31,524 | $40,447 | No |
| #5 | 55 | 75 | Annual | 20 | 30 years of smoking at least 15 cigarettes per day **or** 35 years of smoking at least 10 cigarettes per day (NELSON-like) | No | $71,942,244 | 2,261 | $31,825 | $41,495 | No |
| #6 | 50 | 75 | Annual | 20 | 30 years of smoking at least 15 cigarettes per day **or** 35 years of smoking at least 10 cigarettes per day (NELSON-like) | No | $75,927,044 | 2,354 | $32,259 | $42,794 | No |
| #7 | 50 | 80 | Annual | 20 | 30 years of smoking at least 15 cigarettes per day **or** 35 years of smoking at least 10 cigarettes per day (NELSON-like) | No | $87,643,184 | 2,592 | $33,814 | $49,179 | Yes (Scenario #9) |
| #8 | 50 | 80 | Annual | 20 | 25 years of smoking at least 15 cigarettes per day **or** 30 years of smoking at least 10 cigarettes per day (NELSON-like) | No | $105,283,781 | 2,877 | $36,599 | $61,944 | Yes (Scenario #10) |
| #9 | 50 | 80 | Annual | 20 | 25 years of smoking at least 10 cigarettes per day **or** 30 years of smoking at least 5 cigarettes per day (NELSON-like) | No | $130,052,038 | 3,214 | $40,458 | $73,324 | Yes (Scenario #11) |

**Table G: Cost-effectiveness estimates for lung cancer screening scenarios on the efficient frontier when assuming 50% higher CT examination costs.**

| **Scenario # on current efficient frontier** | **Starting age of screening** | **Stopping age of screening** | **Screening interval** | **Maximum number of years since cessation** | **Cumulative smoking criteria** | **Exclusion from further screening invitations after reaching the maximum number of years since cessation** | **Discounted costs per 100,000** | **Discounted life-years gained per 100,000** | **Costs per life-year gained (discounted) / Average cost-effectiveness ratio (ACER) compared to no screening** | **Incremental cost-effectiveness ratio (ICER) compared to the previous efficient scenario on the current frontier** | **Scenario present on efficient frontier in the base case analyses?** |
| --- | --- | --- | --- | --- | --- | --- | --- | --- | --- | --- | --- |
| #1 | 60 | 75 | Biennial | 10 | 40 pack-years (NLST-like) | Yes | $46,837,102 | 853 | $54,909 | - | No |
| #2 | 55 | 75 | Biennial | 10 | 40 pack-years (NLST-like) | Yes | $55,977,990 | 1,002 | $55,842 | $61,167 | No |
| #3 | 60 | 75 | Annual | 10 | 40 pack-years (NLST-like) | Yes | $74,675,267 | 1,276 | $58,526 | $68,364 | Yes (Scenario #1) |
| #4 | 55 | 75 | Annual | 10 | 40 pack-years (NLST-like) | Yes | $90,366,322 | 1,489 | $60,673 | $73,507 | Yes (Scenario #2) |
| #5 | 55 | 80 | Annual | 10 | 40 pack-years (NLST-like) | Yes | $97,714,197 | 1,564 | $62,474 | $98,372 | No |
| #6 | 55 | 80 | Annual | 10 | 30 pack-years (NLST-like) | Yes | $125,086,809 | 1,834 | $68,209 | $101,463 | Yes (Scenario #4) |
| #7 | 55 | 80 | Annual | 15 | 30 pack-years (NLST-like) | Yes | $155,585,056 | 2,088 | $74,524 | $120,138 | Yes (Scenario #6) |
| #8 | 55 | 80 | Annual | 20 | 30 years of smoking at least 15 cigarettes per day or 35 years of smoking at least 10 cigarettes per day (NELSON-like) | Yes | $189,053,363 | 2,359 | $80,155 | $123,555 | Yes (Scenario #7) |
| #9 | 55 | 80 | Annual | 20 | 30 years of smoking at least 15 cigarettes per day or 35 years of smoking at least 10 cigarettes per day (NELSON-like) | No | $207,607,764 | 2,500 | $83,055 | $131,551 | Yes (Scenario #8) |
| #10 | 50 | 80 | Annual | 20 | 30 years of smoking at least 15 cigarettes per day or 35 years of smoking at least 10 cigarettes per day (NELSON-like) | No | $220,503,567 | 2,592 | $85,075 | $139,816 | Yes (Scenario #9) |
| #11 | 50 | 80 | Annual | 20 | 25 years of smoking at least 15 cigarettes per day or 30 years of smoking at least 10 cigarettes per day (NELSON-like) | No | $278,416,750 | 2,877 | $96,785 | $203,360 | Yes (Scenario #10) |
| #12 | 50 | 80 | Annual | 20 | 25 years of smoking at least 10 cigarettes per day or 30 years of smoking at least 5 cigarettes per day (NELSON-like) | No | $360,005,341 | 3,214 | $111,996 | $241,533 | Yes (Scenario #11) |

**Table H: Presence of the scenarios on the efficient frontier in the base-case analysis across the efficient frontiers of the sensitivity analyses**

| **Scenario number on the efficient frontier in the base-case analysis** | **Scenario present on efficient frontier when assuming high attendance rates?** | **Scenario present on efficient frontier when assuming average attendance rates?** | **Scenario present on efficient frontier when assuming low attendance rates?** | **Scenario present on efficient frontier when assuming 50% lower attributable costs?** | **Scenario present on efficient frontier when assuming 50% higher attributable costs?** | **Scenario present on efficient frontier when assuming 50% lower CT examination costs?** | **Scenario present on efficient frontier when assuming 50% higher CT examination costs?** | **Presence of scenario across the efficient frontiers of the sensitivity analyses (in %)** |
| --- | --- | --- | --- | --- | --- | --- | --- | --- |
| Scenario #1 | No | No | No | Yes | No | No | Yes | 28.57% |
| Scenario #2 | Yes | Yes | No | Yes | Yes | Yes | Yes | 85.71% |
| Scenario #3 | Yes | Yes | Yes | Yes | Yes | Yes | No | 85.71% |
| Scenario #4 | No | No | No | Yes | No | No | Yes | 28.57% |
| Scenario #5 | No | No | No | No | No | No | No | 0.00% |
| Scenario #6 | No | No | No | Yes | No | No | Yes | 28.57% |
| Scenario #7 | No | No | No | Yes | No | No | Yes | 28.57% |
| Scenario #8 | No | No | No | Yes | No | No | Yes | 28.57% |
| Scenario #9 | Yes | Yes | Yes | Yes | Yes | Yes | Yes | 100.00% |
| Scenario #10 | Yes | Yes | Yes | Yes | Yes | Yes | Yes | 100.00% |
| Scenario #11 | Yes | Yes | Yes | Yes | Yes | Yes | Yes | 100.00% |
