# Supplementary material for: Performance and Cost-Effectiveness of Computed Tomography Lung Cancer Screening Scenarios in a Population-Based Setting: A Microsimulation Modeling Analysis in Ontario, Canada
Source: PLoS Med. 2017 Feb 7;14(2):e1002225. doi: 10.1371/journal.pmed.1002225 (PMC5295664; doi:10.1371/journal.pmed.1002225)
Supplement: S6 Text — (DOCX) [file pmed.1002225.s007.docx]

S6. Text: Data Availability Statement

Information on cohort birth tables is available from the Ontario Ministry of Finance (Ontario Populations Projections Update 2011-2036) and from Statisitcs Canada at http://www5.statcan.gc.ca/cansim/a26?lang=eng&retrLang=eng&id=0510001&&pattern=&stByVal=1&p1=1&p2=1&tabMode=dataTable&csid=.

Life-tables by birth year and gender were extracted from the Canadian Human Mortality Database and are available at www.demo.umontreal.ca/chmd/.

Data on age and gender specific lung cancer incidence, histology and stage were obtained from the Ontario Cancer Registry, which can be contacted at ocrquestions@cancercare.on.ca.

The various data on smoking behaviour were derived from the Canadian Community Health Surveys, the National Population Health Surveys, the Canada Health Survey, the General Social Surveys (years 1985&1991) and the Smoking Habits of Canadians Surveys, which are available from Statistics Canada: http://www.statcan.gc.ca/start-debut-eng.html

Data from the National Lung Screening Trial can be requested from the National Cancer Institute’s Cancer Data Access System at https://biometry.nci.nih.gov/cdas/nlst/.

Costing information was obtained from several sources: (1) multiple health services databases maintained by the Ministry of Health and Long Term Care of the province of Ontario, Canada (including hospital care, physician services, drug dispensing, home care, and chronic care) held at the Institute for Clinical Evaluative Sciences (www.ices.on.ca) where data are available to qualified researchers who meet the criteria for access to confidential data, and receive approval from the Research Ethics Board of Sunnybrook Health Sciences Centre; and (2) Cancer Care Ontario, Prevention and Cancer Screening Programs, www.cancercare.on.ca, where data are available to qualified researchers who meet the criteria for data access for projects approved by its Research Ethics Committee.

This work incorporates data analysis provided by the Ontario Tobacco Research Unit, Toronto, Ontario. Parts of this manuscript are based on data and information compiled and provided by the Canadian Institute for Health Information (CIHI). Please see their contact information below.

Canadian Institute for Health Information (CIHI):

4110 Yonge Street, Suite 300

Toronto, Ontario M2P 2B7

Phone: 416-481-2002

Fax: 416-481-2950

www.cihi.ca

Ontario Tobacco Research Unit (College Street Site):

OTRU, c/o ‎Dalla Lana School of Public Health

155 College Street

Toronto, Ontario M5T 3M7

Phone: (416) 978-4538

Fax: (416) 946-0340

Email: info@otru.org
